# Estimates and trends of the global burden of NASH-related liver cancer attributable to high fasting plasma glucose in 1990–2019: analysis of data from the 2019 Global Burden of Disease Study

**DOI:** 10.1186/s13098-022-00976-w

**Published:** 2023-01-17

**Authors:** Ziyi Li, Na Yang, Liyun He, Jialu Wang, Fan Ping, Wei Li, Lingling Xu, Huabing Zhang, Yuxiu Li

**Affiliations:** Department of Endocrinology, Key Laboratory of Endocrinology of National Health Commission, Translation Medicine Center, Peking Union Medical College Hospital, Chinese Academy of Medical Sciences & Peking Union Medical College, No.1 Shuaifuyuan, Wangfujing, Dongcheng District, Beijing, China

**Keywords:** Nonalcoholic steatohepatitis, Liver cancer, High fasting plasma glucose, Global disease burden, Mortality, Disability-adjusted life years

## Abstract

**Background:**

Experimental and epidemiological studies have indicated an association between diabetes exposure and an increased risk of liver cancer due to nonalcoholic steatohepatitis (NASH). However, to date, no systematic study has specifically investigated the burden of NASH-related liver cancer due to exposure to high fasting plasma glucose (HFPG) levels worldwide.

**Methods:**

The number and rate of deaths and disability-adjusted life years (DALYs) from HFPG-induced NASH-related liver cancer were estimated based on the results of the 2019 Global Burden of Disease Study. The estimated annual percentage changes (EAPCs) for age-standardized death or DALYs rates were calculated using a generalized linear model with a Gaussian distribution to quantify the temporal trends in the global burden of NASH-related liver cancer attributable to HFPG. The strength and direction of the association between the sociodemographic index (SDI) and death or DALY rate were measured using Spearman’s rank-order correlation.

**Results:**

Globally, approximately 7.59% of all DALY and 8.76% of all mortalities of NASH-related liver cancer in 2019 were due to HFPG. The age-standardized death and DALY rates of NASH-related liver cancer attributable to HFPG increased from 1990 to 2019. The corresponding EAPCs were 0.69 (95% UI 0.48–0.89), and 0.30 (95% UI 0.05–0.56), respectively. This increasing pattern was most obvious in the high- and low-SDI regions. The age-standardized mortality and DALYs rate of NASH-related liver cancer attributable to HFPG varies considerably worldwide, with the middle SDI region having the highest death and DALY rates in 2019 (DALY 0.96 [95% UI 0.23–2.18]; death 0.05 [95% UI 0.01–0.11]).

**Conclusion:**

The burden of NASH-related liver cancer attributable to HFPG has increased over the past three decades, particularly in regions with high and low SDI.

**Supplementary Information:**

The online version contains supplementary material available at 10.1186/s13098-022-00976-w.

## Introduction

Health priorities in most areas worldwide have fundamentally changed due to the rapid development of medical care and dramatic lifestyle modifications during the last half century [1]. Non-alcoholic fatty liver disease (NAFLD) is a common disease worldwide [2–4]. From a pathological point of view, NAFLD is a progressive disease ranging from steatosis to nonalcoholic steatohepatitis (NASH), liver cirrhosis, hepatocellular carcinoma, liver transplantation, and death [5, 6]. The global prevalence of liver cancer secondary to NASH is increasing. According to a previous study, approximately 15% of hepatocellular carcinoma was secondary to NAFLD and NASH [7]. This staggering burden has led to recent data suggesting that NASH may be the one of the most common drivers of primary liver cancer, with a substantial health economic burden in the near future [8, 9].

Genetics alone cannot explain the substantial increase in the burden of NASH-related liver cancer over the past 30 years or the expected increase in the coming decades [10]. The increasing prevalence of obesity and type 2 diabetes worldwide has induced impairment of glucose and lipid metabolic pathways, which may be one of the most important reasons for the increase in the proportion of NASH-related liver cancer patients [11]. Meanwhile, diabetes has been reported to be independently associated with liver cancer [12]. A recent systematic review indicated that the overall global prevalence of NAFLD and NASH in patients with diabetes may reach 57.80% and 37.33%, respectively [13]. Meanwhile, the risk of liver cancer was two times higher in patients with diabetes mellitus than in those without diabetes mellitus after a median follow-up of 38 months [14]. Recently, the link between NASH-related liver cancer and hyperglycemia was found to be more complex than previously thought. Insulin resistance and the subsequent generation of oxygen free radicals may be important mechanisms for the occurrence and development of liver cancer caused by hyperglycemia [15]. Hyperglycemia is regarded as one of the strongest risk factors for the rapid progression of NAFLD to NASH, advanced fibrosis, and liver cancer [16].

Although NASH-related liver cancer is closely associated with hyperglycemia, its global burden attributable to hyperglycemia has not been well elucidated. Moreover, NASH-related liver cancer burden may vary by region and age. In the present study, we aimed to assess the burden of NASH-related liver cancer attributable to hyperglycemia, using estimates derived from epidemiological studies. We also aimed to evaluate the relative roles of the sociodemographic index (SDI) and age in the observed trend of hyperglycemia-induced NASH-related liver cancer burden.

## Methods

### Data sources

The present study analyzed data obtained from the 2019 Global Burden of Disease (GBD) study. As a continuous quality improvement, GBD has been applying a standard methodological approach to generate estimates for deaths and health loss from several diseases since 1990 and has re-estimated the results every 2–3 years [17, 18]. The GBD 2019 study estimated the disease burden from 286 causes of death, 369 diseases and injuries, and 87 risk factors in 204 countries and territories [19, 20]. An overview of the GBD methodology has been published previously [19]. Briefly, the database of the GBD study comprises a variety of primary data (surveys, censuses, and other health-related data sources). Mortality data were estimated using the cause of death (COD) ensemble model (CODEm), a framework that incorporates statistical models to demonstrate cause-specific death rates [20]. Deaths and disability-adjusted life years (DALYs) were computed as the sum of years of life lost (YLLs) and years lost due to disability (YLDs) for each age, sex, and location [20, 21]. The results were reported according to region, age group, and year. The age-standardized population was calculated using the GBD World Population Age Standard. Mortality and DALY rates were generated from a mean of 1000 draws, and 95% uncertainty intervals (UIs) were computed to estimate the disease burden. The UIs account for not only the variance in parameter estimation but also the uncertainty under the parameter-estimation process, such as data collection and model selection.

For data analysis, the world was divided into 21 regions according to epidemiological similarities and geographical proximity in the 2019 GBD study. In addition, the SDI, which is a composite indicator of the background social and economic conditions that influence health outcomes, was also provided [22]. Countries and territories were classified as quintiles of the SDI (high, high–medium, medium, medium–low, and low level of development).

In the GBD 2019 study, liver cancer was divided into five categories according to etiology: liver cancer due to hepatitis B (GBD code: B.1.7.1), liver cancer due to hepatitis C (GBD code: B.1.7.2), liver cancer due to alcohol use (GBD code: B.1.7.4), liver cancer due to NASH (GBD code: B.1.7.4), and liver cancer due to other causes (GBD code: B.1.7.5). The subtypes of liver cancer were based on the International Classification of Diseases, 10th revision (ICD-10), which is usually determined by a physician’s decision. The subgroups C22-C22.4, C22.7-C22.9, and Z85.05 of ICD-10 were proportionally redistributed into liver cancer due to hepatitis B virus (HBV), hepatitis C virus (HCV), alcohol use, NASH, and other causes [23, 24].

### Risk factors and the definition of high fasting plasma glucose

In the GBD study, all risk factors were divided into three categories: environmental, behavioral, and metabolic risk factors. The risk factors associated with NASH-induced liver cancer include two behavioral risk factors (smoking and drinking) and one metabolic risk factor (high fasting plasma glucose). GBD uses the concept of high fasting plasma glucose (HFPG, defined as any level above the theoretical minimum-risk exposure level [4.8–5.4 mmol/L]) as an individual risk factor to estimate this disease burden [25]. According to the GBD research framework, the disease burden of HFPG is only observed in individuals aged > 25 years.

### Data analysis

Age standardization rates (ASRs) [26], numbers and percentages for mortality, and DALYs were extracted from the GBD 2019 study. The estimated annual percentage changes (EAPCs) [17] for age-standardized mortality (ASMR) or DALYs rates (ASDR) were calculated using a generalized linear model with a Gaussian distribution to quantify the temporal trends in the global burden of NASH-related liver cancer attributable to HFPG [27]. The disease burden increased if the EAPC and lower boundary of its 95% confidence interval (CI) were both > 0. In contrast, a decreasing trend was observed when the EAPC estimation and the upper boundary of its 95% CI were both < 0 [28]. In addition, we assessed the trend of the disease burden in different regions and age groups. The relationship between the ASR and SDI was also evaluated using Spearman’s rank-order correlation.

To explore the influential factors for EAPCs, we assessed the association between EAPCs and ASRs (1990, 2019), the Human Development Index (HDI) (2019), SDI, and the Healthcare Access and Quality Index (HAQI) at the national level using Spearman’s rank order correlation. A hierarchy cluster analysis [17] was conducted to categorize the countries and territories into four groups (a: significant increase, b: increase, c: remained stable or minor decrease, and d: significant decrease) in terms of their temporal trends in ASRs. The population-attributable fraction (PAF) was used to quantify the proportion of cases that could be attributed to risk factors.

Data analysis and illustrations were performed using R software version 4.0.2. The R packages used in our study including “factoextra,” “dplyr,” “tidyverse,” “ggmap,” and “stats.” A p value of less than 0.05 was considered statistically significant.

## Results

### Global DALYs and mortality estimates of HFPG-induced NASH related liver cancer

Approximately 7.59% of all DALY of NASH-related liver cancer cases in 2019 were due to HFPG. The DALY cases increased by 164.5%, from 0.02 million in 1990 to 0.06 million in 2019. Meanwhile, the ASDR of HFPG-induced NASH-related liver cancer increased by 28% (from 0.57 to 0.73 per 100,000 population) with an EAPC of 0.30 (Table 1). As a part of DALYs, the ASR of YLDs increased with an EAPC of 1.11 (95% CI: 0.92–1.29), and the ASR of YLLs increased with an EAPC of 0.29 (95% CI: 0.04–0.55) in the past three decades (Additional file 1: Tables S1 and S2).

**Table 1:** The DALYs of NASH related liver cancer attributable to HFPG in 1990 and 2019, and its temporal trends from 1990 to 2019

Characteristics	1990			2019			1990–2019
	DALYs cases No. (95% UI)	ASR per 1000 No. (95% UI)	Percentage (%)	DALYs cases No. (95% UI)	ASR per 100,000 No. (95% UI)	Percentage (%)	EAPC No. (95% CI)
Overall	22,814.15 (5224.6–51,824.35)	0.57 (0.13–1.31)	4.77 (1.1–10.38)	60,308.72 (14,610.29–135,326.46)	0.73 (0.18–1.65)	7.59 (1.8–16.02)	0.3 (0.05–0.56)
*Socio-demographic index*							
High SDI	3728.39 (884.31–8399.56)	0.35 (0.08–0.8)	6.18 (1.42–13.24)	15,009.74 (3683.96–33,421.49)	0.80 (0.2–1.78)	9.74 (2.39–20.18)	2.87 (2.62–3.11)
High-middle SDI	6188.13 (1408.76–14,086.36)	0.57 (0.13–1.3)	4.98 (1.13–10.86)	9880.89 (2385.02–22,226.78)	0.48 (0.12–1.08)	7.22 (1.69–15.29)	–1.39 (–1.73––1.05)
Middle SDI	9473.12 (2117.33–21,730.86)	0.93 (0.21–2.11)	4.46 (1.03–9.71)	23,847.28 (5768.12–54,084.87)	0.96 (0.23–2.18)	7.18 (1.69–15.15)	–0.61 (–1.05––0.16)
Low-middle SDI	2573.91 (593.05–5825.6)	0.45 (0.1–1.01)	4.27 (0.98–9.29)	8734.82 (2070.69–19,802.48)	0.65 (0.16–1.48)	7.2 (1.7–15.1)	0.81 (0.6–1.03)
Low SDI	837.44 (192.22–2036.17)	0.38 (0.09–0.92)	3.88 (0.88–8.51)	2800.07 (647.08–6650.46)	0.57 (0.14–1.34)	5.64 (1.33–11.87)	1.24 (1.16–1.33)
*Region*							
Andean Latin America	79.78 (16.55–199.66)	0.42 (0.09–1.06)	4.11 (0.86–9.2)	305.12 (66.59–748.71)	0.56 (0.12–1.38)	7.81 (1.67–16.73)	0.68 (0.33–1.03)
Australasia	31.55 (6.75–77.81)	0.13 (0.03–0.32)	3.92 (0.87–8.62)	304.66 (68.48–717.36)	0.61 (0.14–1.44)	6.94 (1.59–14.89)	5.5 (5.07–5.93)
Caribbean	247.02 (56.82–591.37)	0.96 (0.22–2.28)	8.01 (1.85–16.63)	362.26 (88.96–865.62)	0.7 (0.17–1.67)	10.29 (2.49–21.13)	–0.92 (–1.79––0.04)
Central Asia	69.69 (15.19–179.59)	0.15 (0.03–0.4)	3.45 (0.74–7.58)	666.45 (146.5–1639.07)	0.94 (0.21–2.28)	5.97 (1.34–12.74)	5.71 (5.07–6.34)
Central Europe	820.92 (186.76–1900.68)	0.55 (0.13–1.27)	6.49 (1.44–13.92)	1136.53 (272.8–2739.61)	0.52 (0.12–1.24)	9.8 (2.35–20.21)	0.09 (–0.37–0.55)
Central Latin America	435.56 (102.98–1004.28)	0.55 (0.13–1.27)	7.79 (1.83–16.11)	2033.89 (499.42–4618.83)	0.88 (0.22–1.97)	11.7 (2.87–23.56)	1.73 (1.49–1.97)
Central Sub-Saharan Africa	43.87 (9.12–113.7)	0.21 (0.04–0.52)	3.84 (0.85–8.46)	140.87 (29.16–362.18)	0.28 (0.06–0.69)	5.06 (1.14–10.83)	0.8 (0.71–0.9)
East Asia	11,760.54 (2615.07–27,185.61)	1.3 (0.29–2.99)	4.47 (1.02–9.71)	14,381.59 (3385.91–32,233.35)	0.68 (0.16–1.52)	6.05 (1.37–13.15)	–3.72 (–4.39––3.04)
Eastern Europe	238.55 (52.46–548.58)	0.08 (0.02–0.2)	3.38 (0.73–7.39)	835.38 (192.14–1940.18)	0.24 (0.06–0.55)	4.78 (1.04–10.45)	4.19 (3.92–4.45)
Eastern Sub-Saharan Africa	214.5 (47.29–534.27)	0.31 (0.07–0.77)	3.2 (0.72–7.09)	718.23 (157.7–1750.77)	0.48 (0.11–1.18)	4.16 (0.94–9)	1.35 (1.2–1.5)
High-income Asia Pacific	1239.46 (288.87–2772.63)	0.6 (0.14–1.34)	5.41 (1.23–11.74)	3131.43 (737.54–7239.83)	0.69 (0.16–1.62)	7.41 (1.72–16.08)	–0.34 (–0.97–0.29)
High-income North America	1137.48 (267.93–2483.84)	0.32 (0.08–0.69)	7.72 (1.83–16.2)	6915.45 (1756.89–14,784.65)	1.1 (0.28–2.36)	11.82 (2.94–24.03)	5.54 (5.13–5.96)
North Africa and Middle East	1043.71 (230.45–2623.78)	0.64 (0.14–1.59)	4.7 (1.08–10.3)	6237.91 (1407.41–14,658.2)	1.48 (0.34–3.48)	8.88 (2.18–18.68)	3.41 (3.18–3.63)
Oceania	17.22 (3.82–44.07)	0.61 (0.14–1.55)	7.52 (1.82–15.97)	67.6 (16.83–155.11)	1 (0.26–2.32)	12.6 (3.37–25.29)	1.82 (1.65–1.98)
South Asia	1801.6 (411.07–4093.7)	0.34 (0.08–0.77)	4.51 (1.04–9.79)	8282.17 (2018.13–18,376.16)	0.6 (0.15–1.33)	7.93 (1.93–16.45)	1.77 (1.65–1.9)
Southeast Asia	1388.78 (310.64–3365.4)	0.59 (0.13–1.4)	4.08 (0.93–8.91)	7014.6 (1670.42–16,599.05)	1.25 (0.3–3.01)	7.3 (1.71–15.54)	2.52 (2.43–2.62)
Southern Latin America	68.64 (14.72–173.19)	0.15 (0.03–0.37)	5.55 (1.22–11.94)	369.12 (86.57–867.65)	0.44 (0.1–1.02)	9.51 (2.26–19.73)	4.44 (4.25–4.64)
Southern Sub-Saharan Africa	216.97 (45.54–590.34)	0.83 (0.17–2.24)	4.3 (1–9.35)	831.04 (201.54–1831.98)	1.52 (0.37–3.35)	7.02 (1.67–14.79)	1.72 (1.08–2.36)
Tropical Latin America	161.53 (38.59–355.11)	0.18 (0.04–0.4)	5.85 (1.35–12.46)	673.33 (161.98–1460.64)	0.28 (0.07–0.61)	7.76 (1.83–16.27)	2.18 (1.87–2.5)
Western Europe	1475.54 (334.91–3417.45)	0.25 (0.06–0.57)	6.65 (1.54–14.2)	4823.89 (1145.08–11,045.46)	0.53 (0.13–1.21)	9.9 (2.36–20.64)	2.65 (2.45–2.85)
Western Sub-Saharan Africa	321.24 (70.59–783.6)	0.4 (0.09–0.97)	3.35 (0.77–7.42)	1077.22 (236.93–2607.65)	0.65 (0.15–1.54)	4.92 (1.15–10.62)	1.47 (1.34–1.59)

NASH: nonalcoholic steatohepatitis; HFPG: high fasting plasma glucose; DALYs: disability-adjusted life years; EAPCs: Estimated annual percentage changes; ASR: age standardized rate; SDI: socio-demographic index

Globally, nearly 8.76% of all mortalities induced by NASH-related liver cancer in 2019 were due to HFPG. The number of deaths from NASH-related liver cancer attributable to HFPG increased by 196% over the past 30 years, corresponding to an annual percentage change of ASMR of 0.69 from to 1990–2019 (Additional file 1: Table S3).

### HFPG-induced NASH related liver cancer burden by sub-regions

Although the number of DALYs and death cases of HFPG-induced NASH-related liver cancer increased in all sub-regions between 1990 and 2019, ASDR and ASMR did not show a growth pattern in all sub-regions over the same period. For different SDI levels, HFPG-induced NASH related liver cancer burden decreased in middle and high-middle SDI regions (EAPC for ASDR in middle SDI: − 0.61 [− 1.05 to − 0.16]; EAPC for ASMR in middle SDI: − 0.28 [− 0.67 to − 0.11]; EAPC for ASDR in high-middle SDI: − 1.39 [− 1.73 to − 1.05]; EAPC for ASMR in high-middle SDI: − 0.96 [− 1.24 to − 0.68]) (Table 1 and Additional file 1: Table S1).

In 2019, the ASDR and ASMR of HFPG-induced NASH-related liver cancer varied worldwide, with the highest ASRs observed in Southern Sub-Saharan Africa (ASDR: 1.52 [0.37–3.35] per 10,000,000 population; ASMR: 0.08 [0.02–0.17]) per 10,000,000 population), followed by North Africa, the Middle East, and Southeast Asia. The sub-region with the fastest growth in the burden of NASH related liver cancer caused by HFPG was Central Asia (EAPC for ASDR: 5.71 [5.07–6.34]; EAPC for ASMR: 6.06 [5.46–6.66]). Meanwhile, the sub-region with the fastest reduction of the burden of NASH related liver cancer caused by HFPG was East Asia (EAPC for ASDR: − 3.72 [− 4.39 to − 3.04]; EAPC for ASMR: − 3.38 [− 4 to − 2.77]) (Table 1, Additional file 1: Table S1).

### HFPG-induced NASH related liver cancer burden in nations and territories

At national and territorial levels, the burden of NAFLD-related liver cancer varies greatly. Qatar had the highest ASDR and ASMR for NASH-related liver cancer attributable to HFPG in 2019 (ASDR: 10.43 [2.60–23.37], ASMR: 0.63 [0.17–1.41]), followed by Mongolia and Tonga. As for the absolute number, China reported the largest NAFLD-related liver cancer death and DALY cases attributable to HFPG in 2019, followed by India and the United States (Additional file 1: Figures S1, S2, Table S4). According to the results of the cluster analysis, which was measured based on the EPAC of ASRs (Additional file 1: Table S5), 204 countries and territories were categorized into four groups (significant increase group, increase group, remained stable or minor decrease group, and significant decrease group) (Additional file 1: Figure S3).

### Disease burden in different age groups

**Fig. 1:**
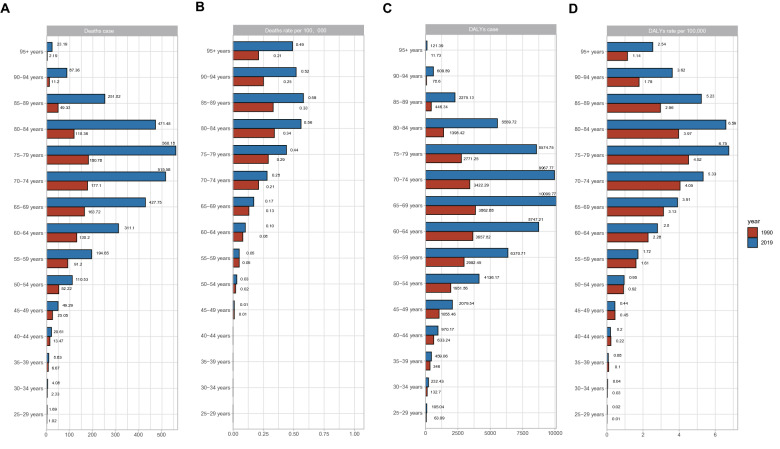
The global disease burden of NASH-related liver cancer attributable to HFPG for different age groups. **A** The deaths number of NASH-related liver cancer attributable to HFPG for different age group in 1990 and 2019; **B** The ASMR of NASH-related liver cancer attributable to HFPG for different age group in 1990 and 2019; **C** The DALYs number of NASH-related liver cancer attributable to HFPG for different age group in 1990 and 2019; **D** The ASDR of NASH-related liver cancer attributable to HFPG for different age group in 1990 and 2019. NASH: nonalcoholic steatohepatitis; ASMR, age-standardized mortality rate; DALYs, disability adjusted life years; ASDR, age-standardized DALYs rate; SDI: socio-demographic index; EPAC: estimated annual percentage changes

In the present study, the age groups with the highest HFPG-induced NAFLD-related liver cancer deaths were those aged 75–79 years, in both 1990 and 2019. The 65–69-year age group had the highest number of DALYs. The 60–84-year age group accounted for most of the overall HFPG-induced NAFLD-related liver cancer death cases (75.21%) in 2019, which was similar to the age distribution of the DALY counts. The DALY and death rates in 2019 increased in nearly all age groups compared with those in 1990. With the increase in age, the growth rate of disease burden increases gradually, with the > 95-year age group having a 130% increase in death rate and a 123% increase in DALY rate (Fig. 1, Additional file 1: Figure S4, S5). The proportion of change in number was significantly higher than the change in rate, and this trend was more obvious as age increased.

### Change of ASRs and PAFs across time and SDI

**Fig. 2:**
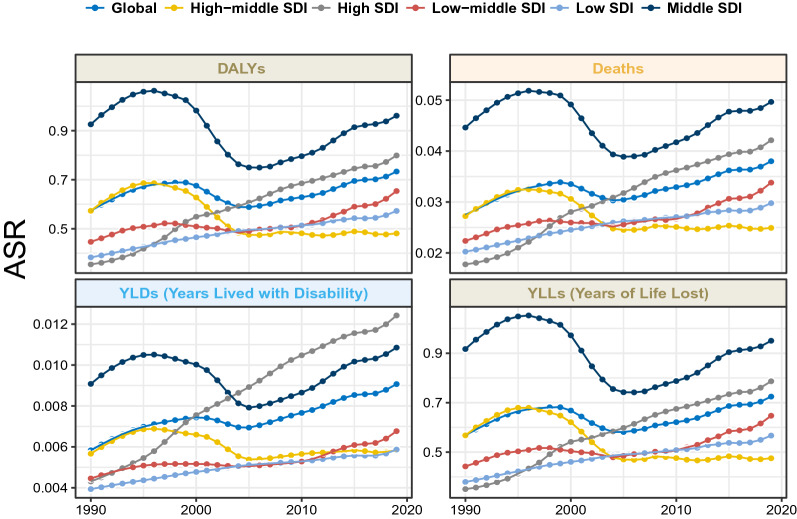
Temporal trend in the deaths, DALYs, YLDs, and YLLs rate of NASH-related liver cancer attributable to HFPG for different SDI regions, 1990–2019. NASH: non-alcoholic fatty liver disease; SDI: socio-demographic index; HFPG: high fasting plasma glucose; ASR, age-standardized rate; DALYs, disability adjusted life years; YLLs: Years of Life Lost; YLDs: Years Lived with Disability; NASH: nonalcoholic steatohepatitis

**Fig. 3:**
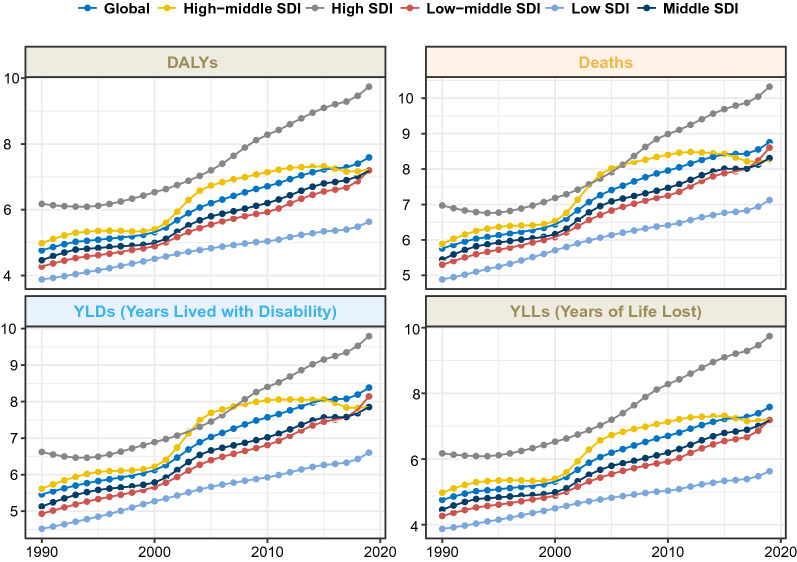
Temporal trend of the PAFs of the NASH-related liver cancer burden attributable to HFPG, 1990–2019. NASH: nonalcoholic steatohepatitis; SDI: socio-demographic index; HFPG: high fasting plasma glucose; DALYs, disability adjusted life years; YLLs: Years of Life Lost; YLDs: Years Lived with Disability. PAF: population attributable fraction

The time trend of NASH-related liver cancer burden varied greatly across the SDI regions. From a global perspective, the ASMR and ASDR of NASH-related liver cancer attributable to HFPG have increased each year, except from 1999 to 2005. The changes in the high and medium SDI regions were consistent with the global change trends. In the high-middle SDI region, the ASMR and ASDR of NASH-related liver cancer attributable to HFPG have recently remained stable (Table 1, Additional file 1: Tables S2–S4, Fig. 2). Globally, the PAFs of HFPG initially increased with time, both worldwide and in different SDI regions. PAFs in the high SDI regions were the highest among the different SDI regions. (Fig. 3).

### Relationship between ASR and SDI

At the regional level, no clear association was found between the SDI and the burden of NASH-related liver cancer attributable to HFPG. However, in general, the burden of HFPG-induced NASH deaths and DALYs increased as the SDI increased in most regions (Additional file 1: Figure S6A, C).

At the country and territorial levels, the HFPG-induced NASH-related liver cancer burden in 2019 slightly increased with increasing socioeconomic development up to an SDI of 0.6, and then eventually decreased (Additional file 1: Figure S6B, D).

### Influential factors for EAPC

**Fig. 4:**
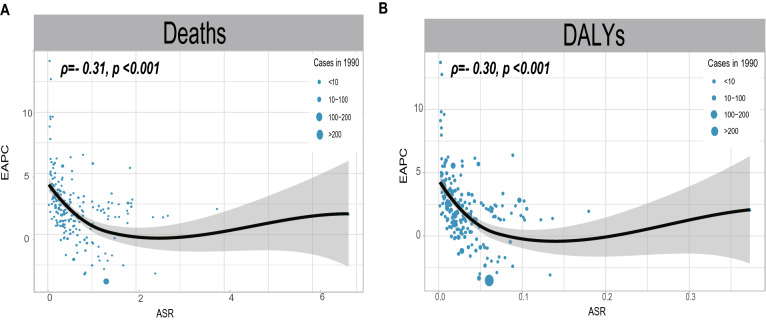
The correlation between EAPC and ASR in 1990. **A** The correlation between EAPC and ASR of NASH-related liver cancer deaths attributable to HFPG in 1990. **B** The correlation between EAPC and ASR of NASH-related liver cancer DALYs in 1990. The circles represent countries that were available on data. The size of circle is increased with the death or DALY cases of NASH-related liver cancer. NASH: nonalcoholic steatohepatitis; HFPG: high fasting plasma glucose; ASR, age-standardized rate; EAPC, estimated annual percentage change

As shown in Fig. 4, a significant negative association was found between EAPCs and ASRs in 1990 (deaths: ρ =  − 0.31, p < 0.001; DALYs: ρ =  − 0.30, p < 0.001). No significant relationship was observed between EAPCs and ASRs in 2019 (p > 0.05) (Additional file 1: Figure S7).

The relationship between EAPCs and the three indicators representing social development level (HDI, SDI, and HAQ) was evaluated. However, only HAQ was negatively correlated with EAPCs (Additional file 1: Figure S8).

## Discussion

With the popularization of HBV vaccination and the implementation of highly effective antiviral therapy for HBV and HCV, the burden of liver cancer caused by HBV and HCV has gradually decreased [24, 29–31]. At the same time, lifestyle changes make NASH the fifth leading cause of primary liver cancer, to which more attention needs to be paid [32]. It is reported that more than one-fifth of NASH patients progress to cirrhosis and 13% directly to liver cancer over 8 years [33, 34]. Furthermore, given that there is still no approved therapy for NASH [35], and that a larger proportion of NASH-related liver cancer is associated with worse survival outcomes than HBV- or HCV-related LC [36]. Therefore, focusing on modifiable risk factors for NASH is of great importance. As a metabolic disease, NASH and its disease burden are closely associated with diabetes. However, previous studies did not evaluate the burden of NASH-related liver cancer caused by diabetes or HFPG. In the present study, the proportion of the burden of NASH-related liver cancer caused by HFPG in the world has gradually increased over the past 30 years. We estimated that approximately 7.59% of all DALYs and 8.76% of all deaths due to NASH-related liver cancer in 2019 were due to HFPG.

Diabetes is involved in the entire process from NAFLD to NASH, and eventually to liver cancer. HFPG-induced insulin resistance (IR) associated with subclinical inflammation is one of the most important causes of NASH-related liver cancer. In the inflammatory state caused by hyperglycemia, the increased flow of free fatty acids (FFA) into the liver leads to fat infiltration in liver cells, which leads to liver damage through lipid peroxidation and mitochondrial dysfunction [37]. In addition, the glucose toxicity caused by persistent hyperglycemia may promote the progress of NASH through glucose-induced increased de novo lipogenesis and hepatocellular dysfunction [38]. Clinical research indicates that the risk of NAFLD increased by 1.21 times per 1 mmol increase in FPG [39], and NASH patients with higher FPG levels were more likely to develop liver cancer [40]. The HFPG pandemic will pose a more serious challenge for NASH-induced liver cancer, which requires necessary measures.

Notably, our study observed socioeconomic differences in the burden of HFPG-induced NASH-related liver cancer. The ASMR and ASDR of NASH-related liver cancer due to HFPG showed an inverted U-shaped relationship with SDI levels, with middle SDI countries having the heaviest burden. In general, a higher SDI is related to better health literacy, sufficient medical resources, and the implementation of preventive measures, which might usually result in a lower disease burden [21, 41, 42]. However, the relationship between SDI and disease burden differs among different diseases. Negative and positive associations were found between the SDI and the global burden of HFPG-induced NCDs and between the SDI and cancer, respectively [43, 44]. In an unhealthy metabolic state, the risk factors for HFPG, such as high body mass index, unhealthy diet, and low physical activity, are growing considerably with the development of SDI [45, 46]. The inconsistency between the HFPG-induced NASH-related liver cancer burden and the SDI can be explained by the failure of health systems to keep pace with the associated population’s health needs.

Although concerns about the HFPG-related burden have historically focused on developed countries [47], our research shows that we may need to pay more attention to rapidly rising developing countries and countries with a rapidly changing lifestyle in the future. Over the past decade, economic development has gradually westernized the lifestyle of developing countries. However, prevention programs for metabolic risk factors in these countries commenced much later than in high-income countries [48]. Implementing a national health strategy is an effective way to reduce the disease burden. Our research showed that although the cases of death and DALY of HFPG-induced NASH-related liver cancer in China are huge, the burden of disease has shown a significant downward trend in the past three decades. Recently, the Chinese government announced ‘Healthy China 2030’, aiming to facilitate appropriate diet and physical activities to reduce obesity and type 2 diabetes in the Chinese population [49]. This kind of national policy will certainly be a breakthrough for reducing HFPG induced NASH-related liver cancer burden in the future.

Advances in medical care appear to have weakened the process by which morbidity translates to an increase in premature mortality over time in most countries. However, the long-term burden of HFPG-induced NASH-related liver cancer has been increasing. Expanding national health systems to provide greater access to evidence-based NASH care could help minimize the burden due to the increasing prevalence.

Additionally, we explored the factors influencing the EAPC of NASH-related liver cancer attributable to HFPG. The amplitude of the ASR variation was significantly negatively associated with the baseline ASR. This may be because of two reasons. First, the lower the baseline ASR, the more significant the ASR variation. Second, countries with low ASR are unlikely to consider the disease a high priority in their prevention programs owing to its limited public health significance compared with other diseases [50]. In addition, the relationship between EAPC and indicators reflecting social development varies with different indicators. This may be related to the evaluation criteria of each indicator.

To our knowledge, this study is the first to comprehensively assess NASH-related liver cancer burden attributable to HFPG by year, age, location, and socioeconomic status using the latest data from the 2019 GBD study, which will be helpful for public health policymakers. Although the GBD estimates fill the gap of sparse or unavailable actual data on disease burden, some limitations should be noted. First, the accuracy of GBD estimates largely depends on the quality and quantity of data used in modeling. However, poor-quality data on the burden of NASH-related liver cancer or HFPG in economically underdeveloped areas might affect the validity of the estimates provided by the model and make wide UIs for some of the estimates. Second, the subtypes or degrees of HFPG, such as type 2 diabetes, type 1 diabetes, and impaired fasting glucose tolerance, were not differentiated. Third, our results were limited to individuals aged > 25 years as children and adolescents were not included. Finally, the burden of NASH-related liver cancer attributed to HFPG was not compared with that attributed to other metabolic risk factors such as body mass index. Notably, most of these limitations were due to the GBD methodology and were beyond the control of researchers.

## Conclusions

Overall, the burden of NASH-related liver cancer attributable to HFPG has increased rapidly in the past three decades, particularly in regions with low and high SDI. Therefore, health authorities and policymakers should urgently increase efforts to stem the growth of HFPG-induced NASH-related liver cancer burden by allocating sufficient resources, developing and implementing diabetes prevention programs, and prioritizing NASH-related liver cancer screening efforts.

## Supplementary Information

**Additional file 1:Table S1.** Deaths of NASH-related liver cancer attributable to HFPG in 1990 and 2019, and its temporal trends from 1990 to 2019.** Table S2.** YLDs of NASH-related liver cancer attributable to HFPG in 1990 and 2019, and its temporal trends from 1990 to 2019.** Table S3.** YLLs of NASH-related liver cancer attributable to HFPG in 1990 and 2019, and its temporal trends from 1990 to 2019.** Table S4.** Deaths and DALYs of NASH-related liver cancer attributable to HFPG in countries and territories.** Table S5.** EAPC of NASH-related liver cancer deaths and DALYs attributable to HPFG in countries and territories.** Figure. S1.** The deaths of NASH-related liver cancer attributable to HFPG in countries and territories. (A) The ASR of NASH-related liver cancer deaths attributable to HFPG in 2019; (B) The relative change in percentage of NASH-related liver cancer deaths attributable to HFPG between 1990 and 2016. ASR, age-standardized rate.** Figure. S2.** The DALYs of NASH-related liver cancer attribute to HFPG in countries and territories. (A) The ASR of DALYs of NASH-related liver cancer attributable to HFPG in 2019; (B) The relative change in percentage of NASH-related liver cancer DALYs attributable to HFPG between 1990 and 2016. ASR, age-standardized rate. DALYs, disability adjusted life years.** Figure. S3.** The clusters of countries and territories in terms of the temporal trends of the NASH-related liver cancer burden attributable to HFPG.** Figure. S4:** Temporal trend of deaths rate of NASH-related liver cancer attributable to HFPG for different age group, 1990–2019.

## Data Availability

The data and research materials supporting the findings of this study are available on the GBD website (https://ghdx.healthdata.org/gbd-2019).

## References

[1] Younossi Z, Anstee QM, Marietti M (2018). Global burden of NAFLD and NASH: trends, predictions, risk factors and prevention. Nat Rev Gastroenterol Hepatol.

[2] Powell EE, Wong VW, Rinella M (2021). Non-alcoholic fatty liver disease. Lancet.

[3] Estes C, Anstee QM, Arias-Loste MT (2018). Modeling NAFLD disease burden in China, France, Germany, Italy, Japan, Spain, United Kingdom, and United States for the period 2016–2030. J Hepatol.

[4] Estes C, Chan HLY, Chien RN (2020). Modelling NAFLD disease burden in four Asian regions-2019-2030. Aliment Pharmacol Ther.

[5] Huang DQ, El-Serag HB, Loomba R (2021). Global epidemiology of NAFLD-related HCC: trends, predictions, risk factors and prevention. Nat Rev Gastroenterol Hepatol.

[6] Paik JM, Golabi P, Younossi Y, Srishord M, Mishra A, Younossi ZM (2020). The growing burden of disability related to nonalcoholic fatty liver disease: data from the global burden of disease 2007–2017. Hepatol Commun.

[7] Tan DJH, Ng CH, Lin SY (2022). Clinical characteristics, surveillance, treatment allocation, and outcomes of non-alcoholic fatty liver disease-related hepatocellular carcinoma: a systematic review and meta-analysis. Lancet Oncol.

[8] Younossi Z, Tacke F, Arrese M (2019). Global perspectives on nonalcoholic fatty liver disease and nonalcoholic steatohepatitis. Hepatology.

[9] Younossi ZM, Stepanova M, Younossi Y (2020). Epidemiology of chronic liver diseases in the USA in the past three decades. Gut.

[10] Diehl AM, Day C (2017). Cause, pathogenesis, and treatment of nonalcoholic steatohepatitis. N Engl J Med.

[11] Targher G, Corey KE, Byrne CD, Roden M (2021). The complex link between NAFLD and type 2 diabetes mellitus - mechanisms and treatments. Nat Rev Gastroenterol Hepatol.

[12] Wong SW, Ting YW, Chan WK (2018). Epidemiology of non-alcoholic fatty liver disease-related hepatocellular carcinoma and its implications. JGH Open.

[13] Younossi ZM, Golabi P, de Avila L (2019). The global epidemiology of NAFLD and NASH in patients with type 2 diabetes: a systematic review and meta-analysis. J Hepatol.

[14] Yang JD, Mohamed HA, Cvinar JL, Gores GJ, Roberts LR, Kim WR (2016). Diabetes mellitus heightens the risk of hepatocellular carcinoma except in patients with hepatitis C cirrhosis. Am J Gastroenterol.

[15] Balkwill F, Mantovani A (2001). Inflammation and cancer: back to Virchow?. Lancet.

[16] Hazlehurst JM, Woods C, Marjot T, Cobbold JF, Tomlinson JW (2016). Non-alcoholic fatty liver disease and diabetes. Metabolism.

[17] Liu Z, Jiang Y, Yuan H (2019). The trends in incidence of primary liver cancer caused by specific etiologies: Results from the Global Burden of Disease Study 2016 and implications for liver cancer prevention. J Hepatol.

[18] Collaborators GBDA (2022). Population-level risks of alcohol consumption by amount, geography, age, sex, and year: a systematic analysis for the Global Burden of Disease Study 2020. Lancet.

[19] Collaborators GBDRF. Global burden of 87 risk factors in 204 countries and territories, 1990–2019: a systematic analysis for the Global Burden of Disease Study 2019. *Lancet.* 2020;396(10258):1223–1249. 10.1016/S0140-6736(20)30752-2 PMC7566194 33069327

[20] Collaborators GBDD (2020). Global age-sex-specific fertility, mortality, healthy life expectancy (HALE), and population estimates in 204 countries and territories, 1950–2019: a comprehensive demographic analysis for the Global Burden of Disease Study 2019. Lancet.

[21] Collaborators GBDUHC. Measuring universal health coverage based on an index of effective coverage of health services in 204 countries and territories, 1990–2019: a systematic analysis for the Global Burden of Disease Study 2019. *Lancet.* 2020;396(10258):1250–1284. 10.1016/S0140-6736(20)30750-9 PMC7562819 32861314

[22] Collaborators GBDCoD. Global, regional, and national age-sex-specific mortality for 282 causes of death in 195 countries and territories, 1980–2017: a systematic analysis for the Global Burden of Disease Study 2017. *Lancet.* 2018;392(10159):1736–1788. 10.1016/S0140-6736(18)32203-7 PMC6227606 30496103

[23] Ghamari SH, Yoosefi M, Abbasi-Kangevari M (2022). Trends in global, regional, and national burden and quality of care index for liver cancer by cause from global burden of Disease 1990–2019. Hepatol Commun.

[24] Liu Y, Zheng J, Hao J (2022). Global burden of primary liver cancer by five etiologies and global prediction by 2035 based on global burden of disease study 2019. Cancer Med.

[25] Liang R, Feng X, Shi D (2022). The global burden of disease attributable to high fasting plasma glucose in 204 countries and territories, 1990–2019: An updated analysis for the Global Burden of Disease Study 2019. Diabetes Metab Res Rev.

[26] Hung GY, Horng JL, Yen HJ, Lee CY, Lin LY (2015). Changing incidence patterns of hepatocellular carcinoma among age groups in Taiwan. J Hepatol.

[27] Li X, Cao X, Guo M, Xie M, Liu X (2020). Trends and risk factors of mortality and disability adjusted life years for chronic respiratory diseases from 1990 to 2017: systematic analysis for the Global Burden of Disease Study 2017. BMJ.

[28] Guerrero-Velasco R, Munoz VH, Concha-Eastman A, Pretel-Meneses AJ, Gutierrez-Martinez MI, Santaella-Tenorio J (2021). Homicide Epidemic in Cali, Colombia: A Surveillance System Data Analysis, 19932018. Am J Public Health.

[29] Xing QQ, Li JM, Dong X (2022). Socioeconomics and attributable etiology of primary liver cancer, 1990–2019. World J Gastroenterol.

[30] Li C, He WQ (2022). Comparison of primary liver cancer mortality estimates from World Health Organization, global burden disease and global cancer observatory. Liver Int.

[31] Sung H, Ferlay J, Siegel RL (2021). Global Cancer Statistics 2020: GLOBOCAN Estimates of Incidence and Mortality Worldwide for 36 Cancers in 185 Countries. CA Cancer J Clin.

[32] Berkan-Kawinska A, Piekarska A (2020). Hepatocellular carcinoma in non-alcohol fatty liver disease - changing trends and specific challenges. Curr Med Res Opin.

[33] Sanyal AJ, Harrison SA, Ratziu V (2019). The natural history of advanced fibrosis due to nonalcoholic steatohepatitis: data from the simtuzumab trials. Hepatology.

[34] Perumpail BJ, Khan MA, Yoo ER, Cholankeril G, Kim D, Ahmed A (2017). Clinical epidemiology and disease burden of nonalcoholic fatty liver disease. World J Gastroenterol.

[35] Sumida Y, Yoneda M (2018). Current and future pharmacological therapies for NAFLD/NASH. J Gastroenterol.

[36] Stine JG, Wentworth BJ, Zimmet A (2018). Systematic review with meta-analysis: risk of hepatocellular carcinoma in non-alcoholic steatohepatitis without cirrhosis compared to other liver diseases. Aliment Pharmacol Ther.

[37] Gastaldelli A, Cusi K (2019). From NASH to diabetes and from diabetes to NASH: mechanisms and treatment options. JHEP Rep.

[38] Luukkonen PK, Sadevirta S, Zhou Y (2018). Saturated fat is more metabolically harmful for the human liver than unsaturated fat or simple sugars. Diabetes Care.

[39] Zou Y, Yu M, Sheng G (2020). Association between fasting plasma glucose and nonalcoholic fatty liver disease in a nonobese Chinese population with normal blood lipid levels: a prospective cohort study. Lipids Health Dis.

[40] Bertot LC, Jeffrey GP, de Boer B (2018). Diabetes impacts prediction of cirrhosis and prognosis by non-invasive fibrosis models in non-alcoholic fatty liver disease. Liver Int.

[41] Xue Y, Zhou J, Wang P (2022). Burden of tuberculosis and its association with socio-economic development status in 204 countries and territories, 1990–2019. Front Med (Lausanne).

[42] Zeng DY, Li JM, Lin S (2021). Global burden of acute viral hepatitis and its association with socioeconomic development status, 1990–2019. J Hepatol.

[43] Safiri S, Nejadghaderi SA, Karamzad N (2022). Global, regional and national burden of cancers attributable to high fasting plasma glucose in 204 countries and territories, 1990–2019. Front Endocrinol (Lausanne).

[44] Ye L, Xu J, Zhang T (2020). Global burden of noncommunicable diseases attributable to high fasting plasma glucose. J Diabetes.

[45] Martinez-Garcia M, Gutierrez-Esparza GO, Roblero-Godinez JC (2021). Cardiovascular risk factors and social development index. Front Cardiovasc Med.

[46] Katikireddi SV, Skivington K, Leyland AH, Hunt K, Mercer SW (2017). The contribution of risk factors to socioeconomic inequalities in multimorbidity across the lifecourse: a longitudinal analysis of the Twenty-07 cohort. BMC Med.

[47] Collaborators GBDDitA. Burden of diabetes and hyperglycaemia in adults in the Americas, 1990–2019: a systematic analysis for the Global Burden of Disease Study 2019. Lancet Diabetes Endocrinol. 2022;10(9):655–667. 10.1016/S2213-8587(22)00186-3 PMC9399220 35850129

[48] Liu J, Bai R, Chai Z, Cooper ME, Zimmet PZ, Zhang L (2022). Low- and middle-income countries demonstrate rapid growth of type 2 diabetes: an analysis based on Global Burden of Disease 1990–2019 data. Diabetologia.

[49] Dong B, Zou Z, Song Y (2020). Adolescent Health and Healthy China 2030: A Review. J Adolesc Health.

[50] Ye F, Zhai M, Long J (2022). The burden of liver cirrhosis in mortality: results from the global burden of disease study. Front Public Health.

