# Supplementary material for: Estimates and trends of the global burden of NASH-related liver cancer attributable to high fasting plasma glucose in 1990–2019: analysis of data from the 2019 Global Burden of Disease Study
Source: Diabetol Metab Syndr. 2023 Jan 17;15:6. doi: 10.1186/s13098-022-00976-w (PMC9843876; doi:10.1186/s13098-022-00976-w)

**Supplementary Material**

**Estimates and trends of the global burden of NASH-related liver cancer attributable to high fasting plasma glucose in 1990–2019: Analysis of data from the 2019 Global Burden of Disease Study**

Table S1. Deaths of NASH-related liver cancer attributable to HFPG in 1990 and 2019, and its temporal trends from 1990 to 2019.

Table S2. YLDs of NASH-related liver cancer attributable to HFPG in 1990 and 2019, and its temporal trends from 1990 to 2019.

Table S3. YLLs of NASH-related liver cancer attributable to HFPG in 1990 and 2019, and its temporal trends from 1990 to 2019.

Table S4. Deaths and DALYs of NASH-related liver cancer attributable to HFPG in countries and territories.

Table S5. EAPC of NASH-related liver cancer deaths and DALYs attributable to HPFG in countries and territories.

Fig. S1. The deaths of NASH-related liver cancer attributable to HFPG in countries and territories. (A) The ASR of NASH-related liver cancer deaths attributable to HFPG in 2019; (B) The relative change in percentage of NASH-related liver cancer deaths attributable to HFPG between 1990 and 2016. ASR, age-standardized rate.

Fig. S2. The DALYs of NASH-related liver cancer attribute to HFPG in countries and territories. (A) The ASR of DALYs of NASH-related liver cancer attributable to HFPG in 2019; (B) The relative change in percentage of NASH-related liver cancer DALYs attributable to HFPG between 1990 and 2016. ASR, age-standardized rate. DALYs, disability adjusted life years.

Fig. S3. The clusters of countries and territories in terms of the temporal trends of the NASH-related liver cancer burden attributable to HFPG.

Fig. S4: Temporal trend of deaths rate of NASH-related liver cancer attributable to HFPG for different age group, 1990–2019.

Fig. S5: Temporal trend of DALYs rate of NASH-related liver cancer attributable to HFPG for different age group, 1990–2019. DALYs, disability adjusted life years.

Fig. S6: Age-standardised rates of the burden of NASH-related liver cancer attributable to HFPG for locations by SDI, 1990–2019. (A) Age-standardised deaths rates of NASH-related liver cancer attributable to HFPG for regions by SDI, 1990–2019. (B) Age-standardised deaths rates of NASH-related liver cancer attributable to HFPG for nations and territories by SDI, 1990–2019. (C) Age-standardised DALYs rates of NASH-related liver cancer attributable to HFPG for regions by SDI, 1990–2019. (B) Age-standardised DALYs rates of NASH-related liver cancer attributable to HFPG for nations and territories by SDI, 1990–2019.

Fig. S7: The correlation between EAPC and ASR in 2019. (A) The correlation between EAPC and ASR of NASH-related liver cancer deaths attributable to HFPG in 2019. (B) The correlation between EAPC and ASR of NASH-related liver cancer DALYs in 2019 The circles represent countries that were available on data. The size of circle is increased with the death or DALY cases of NASH-related liver cancer. NASH: nonalcoholic steatohepatitis; HFPG: high fasting plasma glucose; ASR, age-standardized rate; EAPC, estimated annual percentage change.

Fig. S8: The correlation between EAPC and SDI, HAQ or HDI. (A) The correlation between EAPC and SDI in 2019. (B) The correlation between EAPC and HAQ in 2019. (C) The correlation between EAPC and HDI in 2019 The circles represent countries that were available on data. SDI: socio-demographic index; HAQ: Healthcare Access and Quality; HDI:Human Development Index; EAPCs: Estimated annual percentage changes.

Table S1. The deaths of NAFLD-related liver cancer attributable to HFPG in 1990 and 2019, and its temporal trends from 1990 to 2019.

| Characteristics | 1990 | | | 2019 | | | 1990-2019 |
| --- | --- | --- | --- | --- | --- | --- | --- |
|  | Deaths cases  No. (95% UI) | ASR per 10,000,000  No. (95% UI) | Percentage  (%) | Deaths cases  No. (95% UI) | ASR per 10,000,000  No. (95% UI) | Percentage  (%) | EAPC  No. (95% CI) |
| Overall | 1024.83 (235.19-2285.66) | 0.03 (0.01-0.06) | 5.76 (1.35-12.47) | 3038.14 (743.02-6871.61) | 0.04 (0.01-0.09) | 8.76 (2.09-18.35) | 0.69 (0.48-0.89) |
| Socio-demographic index | | | | | | | |
| High SDI | 190.84 (45.51-427.91) | 0.02 (0-0.04) | 6.97 (1.61-14.76) | 849.79 (207.42-1877.06) | 0.04 (0.01-0.09) | 10.32 (2.52-21.2) | 3.11 (2.84-3.38) |
| High-middle SDI | 279.28 (64.99-623.71) | 0.03 (0.01-0.06) | 5.89 (1.37-12.83) | 507.4 (123.82-1139.99) | 0.02 (0.01-0.06) | 8.26 (1.93-17.3) | -0.96 (-1.24--0.68) |
| Middle SDI | 403.55 (91.23-919.62) | 0.04 (0.01-0.1) | 5.45 (1.27-11.81) | 1134.57 (284.69-2595.82) | 0.05 (0.01-0.11) | 8.31 (1.96-17.47) | -0.28 (-0.67-0.11) |
| Low-middle SDI | 112.68 (26.3-257.04) | 0.02 (0.01-0.05) | 5.3 (1.25-11.47) | 416.16 (98.79-936.74) | 0.03 (0.01-0.08) | 8.61 (2.09-18.01) | 0.98 (0.8-1.16) |
| Low SDI | 37.8 (8.82-90.85) | 0.02 (0-0.05) | 4.89 (1.14-10.64) | 128.37 (30.55-302.87) | 0.03 (0.01-0.07) | 7.12 (1.71-15) | 1.21 (1.11-1.31) |
| Region | | | | | | | |
| Andean Latin America | 4.37 (0.86-11.03) | 0.02 (0-0.06) | 5.23 (1.09-11.56) | 18.18 (3.98-43.74) | 0.03 (0.01-0.08) | 9.18 (2.03-19.5) | 0.91 (0.58-1.25) |
| Australasia | 1.74 (0.38-4.32) | 0.01 (0-0.02) | 4.83 (1.08-10.57) | 17.99 (4.05-43.41) | 0.03 (0.01-0.08) | 8.03 (1.86-17) | 5.59 (5.09-6.09) |
| Caribbean | 13.2 (3.08-31.62) | 0.05 (0.01-0.13) | 9.54 (2.23-19.73) | 19.63 (4.84-45.85) | 0.04 (0.01-0.09) | 11.88 (3.01-24.26) | -0.9 (-1.8-0) |
| Central Asia | 3.2 (0.68-8.12) | 0.01 (0-0.02) | 3.93 (0.85-8.62) | 29.5 (6.65-72.44) | 0.05 (0.01-0.11) | 6.78 (1.54-14.41) | 6.06 (5.46-6.66) |
| Central Europe | 43.51 (10.02-99.07) | 0.03 (0.01-0.07) | 7.33 (1.62-15.65) | 63.53 (15.28-150.18) | 0.03 (0.01-0.07) | 10.65 (2.54-21.99) | -0.02 (-0.52-0.48) |
| Central Latin America | 21.9 (5.21-49.59) | 0.03 (0.01-0.07) | 10.03 (2.38-20.56) | 104 (26.14-236.08) | 0.05 (0.01-0.1) | 13.28 (3.32-26.68) | 1.6 (1.35-1.85) |
| Central Sub-Saharan Africa | 1.84 (0.39-4.77) | 0.01 (0-0.03) | 5.06 (1.15-11.06) | 5.94 (1.29-14.76) | 0.01 (0-0.03) | 6.69 (1.59-13.91) | 0.74 (0.66-0.83) |
| East Asia | 480.94 (106.68-1099.77) | 0.06 (0.01-0.14) | 5.3 (1.22-11.56) | 660.89 (153.38-1486.72) | 0.03 (0.01-0.07) | 6.76 (1.53-14.64) | -3.38 (-4--2.77) |
| Eastern Europe | 11.8 (2.54-26.98) | 0 (0-0.01) | 3.91 (0.84-8.64) | 44.38 (9.97-102.71) | 0.01 (0-0.03) | 5.43 (1.19-11.84) | 4.25 (4.01-4.49) |
| Eastern Sub-Saharan Africa | 9.92 (2.25-24.59) | 0.02 (0-0.04) | 4.14 (0.95-9.2) | 33.84 (7.59-81.64) | 0.03 (0.01-0.06) | 5.37 (1.24-11.55) | 1.36 (1.21-1.5) |
| High-income Asia Pacific | 56.91 (13.27-128.1) | 0.03 (0.01-0.06) | 5.93 (1.4-12.85) | 195.98 (44.69-453.53) | 0.04 (0.01-0.09) | 7.77 (1.81-16.45) | 0.22 (-0.39-0.84) |
| High-income North America | 61.14 (14.71-132.55) | 0.02 (0-0.04) | 8.47 (1.99-17.75) | 364.03 (91.62-783.18) | 0.06 (0.01-0.12) | 12.61 (3.21-25.6) | 5.49 (5.07-5.92) |
| North Africa and Middle East | 47.41 (10.68-116.62) | 0.03 (0.01-0.08) | 5.7 (1.33-12.33) | 279.94 (65.47-656.86) | 0.07 (0.02-0.17) | 10.33 (2.52-21.51) | 3.26 (3.03-3.48) |
| Oceania | 0.72 (0.17-1.82) | 0.03 (0.01-0.07) | 8.96 (2.21-18.53) | 2.8 (0.71-6.47) | 0.05 (0.01-0.12) | 14.26 (3.8-28.19) | 1.75 (1.63-1.88) |
| South Asia | 78.51 (18.02-177.77) | 0.02 (0-0.04) | 5.58 (1.29-11.99) | 390.3 (94.13-862.43) | 0.03 (0.01-0.07) | 9.41 (2.29-19.47) | 1.78 (1.62-1.94) |
| Southeast Asia | 66.15 (14.97-156.41) | 0.03 (0.01-0.07) | 5.32 (1.26-11.49) | 361.14 (86.53-870.74) | 0.07 (0.02-0.17) | 8.86 (2.13-18.78) | 2.81 (2.69-2.93) |
| Southern Latin America | 3.65 (0.78-9.18) | 0.01 (0-0.02) | 6.34 (1.4-13.48) | 20.82 (4.92-49.33) | 0.02 (0.01-0.06) | 10.42 (2.48-21.69) | 4.51 (4.3-4.73) |
| Southern Sub-Saharan Africa | 10.83 (2.25-29.25) | 0.05 (0.01-0.13) | 6.11 (1.44-13.07) | 39.14 (9.63-85.53) | 0.08 (0.02-0.17) | 9.04 (2.15-18.86) | 1.46 (0.83-2.1) |
| Tropical Latin America | 7.59 (1.8-16.64) | 0.01 (0-0.02) | 7.34 (1.72-15.45) | 34.81 (8.42-75.5) | 0.02 (0-0.03) | 9.15 (2.15-19.2) | 2.26 (1.93-2.6) |
| Western Europe | 83.51 (18.94-195.15) | 0.01 (0-0.03) | 7.51 (1.7-15.97) | 298.09 (69.39-688.57) | 0.03 (0.01-0.07) | 10.84 (2.59-22.32) | 2.77 (2.52-3.01) |
| Western Sub-Saharan Africa | 16.00 (3.52-38.93) | 0.02 (0-0.05) | 4.36 (1-9.56) | 53.19 (11.95-125.45) | 0.04 (0.01-0.08) | 6.35 (1.51-13.61) | 1.48 (1.33-1.63) |

NASH: nonalcoholic steatohepatitis; HFPG: high fasting plasma glucose; EAPCs: Estimated annual percentage changes; ASR: age standardized rate; SDI: socio-demographic index.

Table S2. YLDs of NASH-related liver cancer attributable to HFPG in 1990 and 2019, and its temporal trends from 1990 to 2019.

| Characteristics | 1990 | | | 2019 | | | 1990-2019 |
| --- | --- | --- | --- | --- | --- | --- | --- |
|  | YLDs cases  No (95% UI) | ASR per 100,000  No. (95% UI) | Percentage  (%) | YLDs cases  No (95% UI) | ASR per 100,000  No. (95% UI) | Percentage  (%) | EAPC  No. (95% CI) |
| Over all | 223.07 (48.38-537.91) | 0.01 (0-0.01) | 5.46 (1.24-11.77) | 732.4 (173.25-1755.62) | 0.01 (0-0.02) | 8.38 (1.98-17.57) | 1.11 (0.92-1.29) |
| Socio-demographic index | | | | | | | |
| High SDI | 46.02 (10.37-111.41) | 0 (0-0.01) | 6.62 (1.53-14.14) | 243.62 (57.51-577.91) | 0.01 (0-0.03) | 9.79 (2.39-20.41) | 3.75 (3.43-4.07) |
| High-middle SDI | 59.43 (13.17-141.59) | 0.01 (0-0.01) | 5.61 (1.29-12.08) | 119.36 (27.67-287.45) | 0.01 (0-0.01) | 7.86 (1.84-16.58) | -0.55 (-0.82--0.28) |
| Middle SDI | 86.07 (18.48-206.15) | 0.01 (0-0.02) | 5.13 (1.18-11.12) | 256.51 (58.55-615.79) | 0.01 (0-0.03) | 7.85 (1.84-16.52) | -0.06 (-0.47-0.35) |
| Low-middle SDI | 23.62 (5.35-58.55) | 0 (0-0.01) | 4.93 (1.12-10.61) | 86.02 (20-205.61) | 0.01 (0-0.02) | 8.14 (1.94-17.07) | 0.98 (0.8-1.17) |
| Low SDI | 7.79 (1.68-19.92) | 0 (0-0.01) | 4.52 (1.04-9.89) | 26.47 (5.99-66.05) | 0.01 (0-0.01) | 6.6 (1.56-13.99) | 1.25 (1.16-1.34) |
| Region | | | | | | | |
| Andean Latin America | 0.85 (0.16-2.23) | 0 (0-0.01) | 4.84 (1.02-10.77) | 3.49 (0.69-8.94) | 0.01 (0-0.02) | 8.73 (1.91-18.41) | 0.85 (0.51-1.2) |
| Australasia | 0.37 (0.07-0.97) | 0 (0-0) | 4.45 (0.98-9.75) | 4.18 (0.87-10.76) | 0.01 (0-0.02) | 7.54 (1.75-16.1) | 5.95 (5.39-6.52) |
| Caribbean | 2.64 (0.55-6.69) | 0.01 (0-0.03) | 9.05 (2.12-18.85) | 4 (0.88-10.09) | 0.01 (0-0.02) | 11.43 (2.88-23.13) | -0.77 (-1.6-0.07) |
| Central Asia | 0.66 (0.13-1.73) | 0 (0-0) | 3.78 (0.8-8.3) | 6.19 (1.26-15.84) | 0.01 (0-0.02) | 6.58 (1.49-14.01) | 5.86 (5.26-6.47) |
| Central Europe | 8.68 (1.8-21.17) | 0.01 (0-0.01) | 7.07 (1.56-15.15) | 13.15 (2.89-32.47) | 0.01 (0-0.01) | 10.42 (2.47-21.57) | 0.27 (-0.19-0.73) |
| Central Latin America | 4.42 (0.96-10.45) | 0.01 (0-0.01) | 9.25 (2.18-19.11) | 21.29 (5.12-50.25) | 0.01 (0-0.02) | 12.74 (3.16-25.49) | 1.77 (1.53-2) |
| Central Sub-Saharan Africa | 0.39 (0.08-1.05) | 0 (0-0.01) | 4.55 (1.01-9.94) | 1.25 (0.26-3.26) | 0 (0-0.01) | 6.04 (1.37-12.71) | 0.81 (0.72-0.9) |
| East Asia | 104.36 (22.33-251.5) | 0.01 (0-0.03) | 5.04 (1.14-10.91) | 162.89 (35.3-395.09) | 0.01 (0-0.02) | 6.49 (1.48-14.03) | -2.89 (-3.51--2.28) |
| Eastern Europe | 2.41 (0.5-5.86) | 0 (0-0) | 3.73 (0.8-8.23) | 9.11 (1.93-22.27) | 0 (0-0.01) | 5.23 (1.14-11.47) | 4.34 (4.1-4.58) |
| Eastern Sub-Saharan Africa | 2.02 (0.42-5.26) | 0 (0-0.01) | 3.79 (0.86-8.37) | 6.82 (1.49-17.32) | 0.01 (0-0.01) | 4.96 (1.16-10.68) | 1.31 (1.17-1.46) |
| High-income Asia Pacific | 16.18 (3.6-38.88) | 0.01 (0-0.02) | 5.72 (1.33-12.41) | 70.41 (15.7-172.31) | 0.01 (0-0.04) | 7.59 (1.77-16.06) | 1.27 (0.75-1.78) |
| High-income North America | 14.01 (3.25-33) | 0 (0-0.01) | 8.17 (1.93-17.14) | 95.87 (22.96-219.94) | 0.02 (0-0.03) | 12.17 (3.04-24.93) | 5.97 (5.54-6.4) |
| North Africa and Middle East | 9.98 (2.1-25.96) | 0.01 (0-0.02) | 5.36 (1.24-11.62) | 67.6 (14.96-165.26) | 0.02 (0-0.04) | 9.89 (2.42-20.42) | 3.83 (3.56-4.1) |
| Oceania | 0.15 (0.03-0.41) | 0.01 (0-0.02) | 8.43 (2.07-17.7) | 0.61 (0.14-1.48) | 0.01 (0-0.03) | 13.68 (3.65-27.15) | 1.83 (1.69-1.97) |
| South Asia | 16.48 (3.73-39.69) | 0 (0-0.01) | 5.18 (1.2-11.18) | 80.68 (18.95-189.07) | 0.01 (0-0.01) | 8.93 (2.19-18.54) | 1.84 (1.71-1.97) |
| Southeast Asia | 13.52 (2.9-35.14) | 0.01 (0-0.02) | 4.88 (1.14-10.58) | 76.08 (16.9-194.03) | 0.01 (0-0.04) | 8.31 (1.99-17.64) | 2.89 (2.8-2.98) |
| Southern Latin America | 0.73 (0.14-1.93) | 0 (0-0) | 6.1 (1.33-12.93) | 4.28 (0.92-11.26) | 0 (0-0.01) | 10.13 (2.4-21.04) | 4.66 (4.44-4.88) |
| Southern Sub-Saharan Africa | 2.18 (0.43-6.17) | 0.01 (0-0.02) | 5.47 (1.27-11.72) | 8.08 (1.86-19.04) | 0.02 (0-0.04) | 8.4 (1.97-17.49) | 1.59 (1-2.19) |
| Tropical Latin America | 1.56 (0.35-3.71) | 0 (0-0) | 6.83 (1.59-14.41) | 7.07 (1.67-16.48) | 0 (0-0.01) | 8.67 (2-18.24) | 2.3 (1.97-2.64) |
| Western Europe | 18.27 (3.88-44.84) | 0 (0-0.01) | 7.17 (1.64-15.28) | 78.66 (17.35-192.62) | 0.01 (0-0.02) | 10.51 (2.5-21.67) | 3.57 (3.25-3.88) |
| Western Sub-Saharan Africa | 3.2 (0.66-8.29) | 0 (0-0.01) | 4 (0.91-8.78) | 10.69 (2.3-26.67) | 0.01 (0-0.02) | 5.87 (1.37-12.54) | 1.49 (1.36-1.63) |

NASH: nonalcoholic steatohepatitis; HFPG: high fasting plasma glucose; YLDs: Years Lived with Disability; EAPCs: Estimated annual percentage changes; ASR: age standardized rate; SDI: socio-demographic index.

Table S3. YLDs of NASH-related liver cancer attributable to HFPG in 1990 and 2019, and its temporal trends from 1990 to 2019.

| Characteristics | 1990 | | | 2019 | | | 1990-2019 |
| --- | --- | --- | --- | --- | --- | --- | --- |
|  | YLLs cases  No (95% UI) | ASR per 100,000  No. (95% UI) | Percentage  (%) | YLLs cases  No (95% UI) | ASR per 100,000  No. (95% UI) | Percentage  (%) | EAPC  No. (95% CI) |
| Over all | 22591.08 (5177.96-51267.03) | 0.57 (0.13-1.3) | 4.76 (1.1-10.36) | 59576.32 (14474.73-133547.01) | 0.72 (0.18-1.63) | 7.58 (1.8-16) | 0.29 (0.04-0.55) |
| Socio-demographic index | | | | | | | |
| High SDI | 3682.38 (872.62-8295.23) | 0.35 (0.08-0.79) | 6.17 (1.42-13.23) | 14766.12 (3623.3-32777.95) | 0.79 (0.19-1.75) | 9.74 (2.39-20.19) | 2.86 (2.61-3.1) |
| High-middle SDI | 6128.7 (1393.83-13957.81) | 0.57 (0.13-1.29) | 4.98 (1.13-10.85) | 9761.53 (2349.26-21989.02) | 0.48 (0.11-1.07) | 7.21 (1.68-15.28) | -1.4 (-1.74--1.06) |
| Middle SDI | 9387.06 (2101.08-21514.27) | 0.92 (0.21-2.09) | 4.46 (1.03-9.69) | 23590.76 (5711.7-53531.48) | 0.95 (0.23-2.16) | 7.18 (1.68-15.14) | -0.61 (-1.05--0.17) |
| Low-middle SDI | 2550.28 (588.74-5779.16) | 0.44 (0.1-1) | 4.27 (0.98-9.28) | 8648.8 (2050.98-19648.64) | 0.65 (0.15-1.46) | 7.19 (1.7-15.08) | 0.81 (0.6-1.03) |
| Low SDI | 829.65 (190.6-2018.85) | 0.38 (0.09-0.9) | 3.88 (0.88-8.5) | 2773.61 (642.09-6602.62) | 0.57 (0.13-1.32) | 5.63 (1.33-11.85) | 1.24 (1.16-1.33) |
| Region | | | | | | | |
| Andean Latin America | Andean Latin America | 78.93 (16.36-197.09) | 0.42 (0.09-1.05) | 4.1 (0.86-9.19) | 301.62 (65.96-738.38) | 0.56 (0.12-1.36) | 7.8 (1.67-16.71) |
| Australasia | Australasia | 31.18 (6.69-76.91) | 0.13 (0.03-0.32) | 3.92 (0.87-8.61) | 300.48 (67.61-709.96) | 0.6 (0.14-1.43) | 6.94 (1.59-14.88) |
| Caribbean | Caribbean | 244.38 (56.32-583.85) | 0.95 (0.22-2.25) | 8 (1.85-16.61) | 358.26 (87.87-858.44) | 0.69 (0.17-1.66) | 10.28 (2.49-21.12) |
| Central Asia | Central Asia | 69.03 (15.08-177.57) | 0.15 (0.03-0.39) | 3.45 (0.74-7.57) | 660.25 (145.38-1630.78) | 0.93 (0.21-2.26) | 5.97 (1.34-12.72) |
| Central Europe | Central Europe | 812.25 (185.13-1880.76) | 0.55 (0.13-1.26) | 6.48 (1.44-13.91) | 1123.37 (269.47-2709.87) | 0.51 (0.12-1.23) | 9.79 (2.34-20.19) |
| Central Latin America | Central Latin America | 431.14 (101.91-991.62) | 0.55 (0.13-1.25) | 7.77 (1.83-16.09) | 2012.6 (492.42-4572.44) | 0.87 (0.22-1.96) | 11.69 (2.87-23.55) |
| Central Sub-Saharan Africa | Central Sub-Saharan Africa | 43.48 (9.03-113.15) | 0.2 (0.04-0.52) | 3.83 (0.85-8.45) | 139.62 (28.94-359.22) | 0.28 (0.06-0.69) | 5.05 (1.14-10.82) |
| East Asia | East Asia | 11656.17 (2588.84-26902.75) | 1.29 (0.29-2.97) | 4.47 (1.02-9.7) | 14218.7 (3354.75-31822.73) | 0.67 (0.16-1.51) | 6.05 (1.37-13.14) |
| Eastern Europe | Eastern Europe | 236.14 (51.89-543.57) | 0.08 (0.02-0.19) | 3.38 (0.73-7.39) | 826.27 (190.08-1916.04) | 0.24 (0.05-0.55) | 4.78 (1.04-10.44) |
| Eastern Sub-Saharan Africa | Eastern Sub-Saharan Africa | 212.48 (46.71-528.71) | 0.31 (0.07-0.76) | 3.19 (0.72-7.08) | 711.41 (156.32-1731.64) | 0.48 (0.11-1.16) | 4.15 (0.94-8.98) |
| High-income Asia Pacific | High-income Asia Pacific | 1223.28 (285.68-2737.79) | 0.59 (0.14-1.32) | 5.41 (1.23-11.74) | 3061.02 (720.64-7086.29) | 0.68 (0.16-1.59) | 7.41 (1.72-16.07) |
| High-income North America | High-income North America | 1123.46 (264.54-2452.86) | 0.32 (0.07-0.68) | 7.72 (1.83-16.19) | 6819.59 (1732.98-14610.17) | 1.08 (0.28-2.33) | 11.82 (2.94-24.02) |
| North Africa and Middle East | North Africa and Middle East | 1033.73 (228.65-2593.97) | 0.63 (0.14-1.57) | 4.7 (1.08-10.29) | 6170.31 (1392.72-14488.26) | 1.46 (0.34-3.44) | 8.87 (2.18-18.66) |
| Oceania | Oceania | 17.06 (3.79-43.7) | 0.61 (0.14-1.53) | 7.52 (1.82-15.95) | 66.99 (16.68-153.88) | 0.99 (0.25-2.3) | 12.59 (3.37-25.27) |
| South Asia | South Asia | 1785.11 (407.41-4057) | 0.34 (0.08-0.76) | 4.5 (1.04-9.77) | 8201.48 (1992.23-18192.55) | 0.6 (0.14-1.32) | 7.92 (1.93-16.43) |
| Southeast Asia | Southeast Asia | 1375.26 (308.11-3329.51) | 0.58 (0.13-1.39) | 4.07 (0.93-8.9) | 6938.52 (1653.3-16433.5) | 1.23 (0.3-2.98) | 7.29 (1.71-15.52) |
| Southern Latin America | Southern Latin America | 67.91 (14.54-171.34) | 0.15 (0.03-0.37) | 5.55 (1.22-11.93) | 364.84 (85.52-856.25) | 0.43 (0.1-1.01) | 9.5 (2.26-19.72) |
| Southern Sub-Saharan Africa | Southern Sub-Saharan Africa | 214.8 (45.12-584.38) | 0.82 (0.17-2.22) | 4.29 (1-9.34) | 822.96 (199.48-1815.16) | 1.51 (0.36-3.32) | 7.01 (1.67-14.77) |
| Tropical Latin America | Tropical Latin America | 159.96 (38.29-351.78) | 0.18 (0.04-0.4) | 5.84 (1.35-12.44) | 666.26 (160.27-1446.12) | 0.28 (0.07-0.6) | 7.75 (1.83-16.26) |
| Western Europe | Western Europe | 1457.27 (330.35-3378.25) | 0.24 (0.06-0.56) | 6.64 (1.54-14.19) | 4745.23 (1127.77-10825.63) | 0.52 (0.12-1.19) | 9.89 (2.35-20.62) |
| Western Sub-Saharan Africa | Western Sub-Saharan Africa | 318.04 (69.99-776.22) | 0.4 (0.09-0.97) | 3.35 (0.76-7.4) | 1066.53 (234.9-2584.02) | 0.64 (0.15-1.52) | 4.91 (1.15-10.61) |

NASH: nonalcoholic steatohepatitis; HFPG: high fasting plasma glucose; YLLs: Years of Life Lost; EAPCs: Estimated annual percentage changes; ASR: age standardized rate, SDI: socio-demographic index.

Table S4 Deaths and DALYs of NASH-related liver cancer attributable to HFPG in countries and territories.

| location | Deaths case change from 1990 to 2019（%） | Deaths ASR in 2019 | Deaths case in 2019 No. 10^3^ (95% UI) | DALYs case change from 1990 to 2019（%） | DALYs ASR in 2019 | DALYs case in 2019 No. 10^3^ (95% UI) |
| --- | --- | --- | --- | --- | --- | --- |
| Afghanistan | 4.187 | 0.114 (0.027-0.279) | 11.772 (2.756-28.983) | 99.391 | 2.443 (0.579-6.053) | 298.700 (69.549-758.532) |
| Albania | 0.667 | 0.036 (0.007-0.093) | 1.585 (0.327-4.172) | 12.858 | 0.645 (0.138-1.708) | 28.621 (6.086-76.083) |
| Algeria | 1.24 | 0.033 (0.008-0.078) | 9.641 (2.177-22.416) | 25.649 | 0.6 (0.136-1.413) | 191.435 (42.751-461.241) |
| American Samoa | 0.018 | 0.175 (0.047-0.389) | 0.078 (0.021-0.173) | 0.425 | 3.79 (0.968-8.462) | 1.868 (0.467-4.208) |
| Andorra | 0.014 | 0.055 (0.011-0.143) | 0.078 (0.016-0.203) | 0.267 | 0.968 (0.199-2.463) | 1.342 (0.278-3.403) |
| Angola | 0.29 | 0.015 (0.003-0.036) | 1.241 (0.265-3.126) | 6.986 | 0.284 (0.061-0.712) | 29.43 (6.25-75.459) |
| Antigua and Barbuda | 0.036 | 0.04 (0.01-0.093) | 0.037 (0.009-0.086) | 0.641 | 0.71 (0.171-1.69) | 0.7 (0.165-1.688) |
| Argentina | 2.312 | 0.019 (0.004-0.047) | 10.812 (2.455-26.324) | 42.253 | 0.342 (0.08-0.819) | 188.375 (43.907-452.859) |
| Armenia | 0.085 | 0.052 (0.011-0.125) | 2.13 (0.456-5.167) | 1.746 | 0.942 (0.202-2.264) | 39.474 (8.403-95.232) |
| Australia | 1.452 | 0.035 (0.008-0.086) | 15.71 (3.495-38.438) | 25.84 | 0.627 (0.14-1.501) | 264.044 (57.34-634.792) |
| Austria | 0.853 | 0.019 (0.004-0.048) | 3.686 (0.809-9.218) | 14.953 | 0.345 (0.075-0.862) | 62.012 (13.324-155.923) |
| Azerbaijan | 0.129 | 0.032 (0.007-0.08) | 2.335 (0.473-5.796) | 2.635 | 0.593 (0.121-1.489) | 51.344 (10.317-128.874) |
| Bahamas | 0.082 | 0.039 (0.009-0.096) | 0.137 (0.032-0.338) | 1.722 | 0.757 (0.177-1.892) | 2.902 (0.669-7.313) |
| Bahrain | 0.12 | 0.18 (0.048-0.391) | 1.017 (0.257-2.265) | 2.545 | 3.006 (0.78-6.558) | 22.679 (5.421-50.961) |
| Bangladesh | 4.396 | 0.016 (0.004-0.04) | 19.177 (4.292-47.369) | 98.987 | 0.298 (0.067-0.731) | 382.472 (85.178-955.747) |
| Barbados | 0.089 | 0.038 (0.009-0.091) | 0.194 (0.047-0.46) | 1.488 | 0.689 (0.166-1.649) | 3.461 (0.829-8.311) |
| Belarus | 0.491 | 0.009 (0.002-0.023) | 1.425 (0.28-3.701) | 9.366 | 0.166 (0.033-0.436) | 26.748 (5.253-71.087) |
| Belgium | 1.722 | 0.021 (0.005-0.052) | 5.27 (1.169-13.038) | 28.118 | 0.372 (0.085-0.914) | 85.125 (19.454-206.947) |
| Belize | 0.035 | 0.034 (0.008-0.082) | 0.082 (0.019-0.199) | 0.663 | 0.648 (0.15-1.575) | 1.706 (0.391-4.161) |
| Benin | 0.512 | 0.045 (0.009-0.116) | 1.786 (0.378-4.609) | 9.935 | 0.816 (0.175-2.131) | 35.854 (7.791-94.694) |
| Bermuda | 0.029 | 0.021 (0.005-0.053) | 0.03 (0.007-0.075) | 0.526 | 0.361 (0.079-0.877) | 0.486 (0.107-1.191) |
| Bhutan | 0.018 | 0.028 (0.006-0.071) | 0.142 (0.029-0.366) | 0.425 | 0.522 (0.108-1.351) | 2.84 (0.584-7.393) |
| Bolivia (Plurinational State of) | 0.816 | 0.052 (0.011-0.128) | 3.968 (0.829-10.062) | 15.915 | 0.865 (0.179-2.222) | 71.834 (14.781-187.389) |
| Bosnia and Herzegovina | 0.999 | 0.101 (0.023-0.247) | 6.182 (1.44-15.337) | 21.493 | 1.808 (0.424-4.437) | 112.141 (26.269-275.617) |
| Botswana | 0.028 | 0.016 (0.004-0.04) | 0.179 (0.039-0.443) | 0.608 | 0.315 (0.068-0.788) | 4.058 (0.857-10.071) |
| Brazil | 7.435 | 0.015 (0.004-0.033) | 34.195 (8.19-74.259) | 158.48 | 0.282 (0.067-0.61) | 661.638 (158.749-1432.645) |
| Brunei Darussalam | 0.07 | 0.131 (0.034-0.299) | 0.301 (0.076-0.692) | 1.528 | 2.494 (0.641-5.791) | 7.165 (1.733-16.642) |
| Bulgaria | 3.657 | 0.03 (0.007-0.075) | 4.677 (1.087-11.6) | 75.547 | 0.591 (0.131-1.485) | 86.764 (19.779-216.537) |
| Burkina Faso | 0.439 | 0.016 (0.004-0.04) | 1.158 (0.25-2.843) | 8.877 | 0.291 (0.061-0.716) | 23.885 (4.831-58.763) |
| Burundi | 0.398 | 0.022 (0.004-0.059) | 0.759 (0.153-2.046) | 8.442 | 0.412 (0.083-1.114) | 16.952 (3.269-46.717) |
| Cabo Verde | 0.016 | 0.15 (0.034-0.366) | 0.585 (0.131-1.401) | 0.266 | 2.544 (0.586-6.215) | 9.94 (2.287-24.134) |
| Cambodia | 0.96 | 0.062 (0.013-0.149) | 6.202 (1.39-15.043) | 22.142 | 1.166 (0.259-2.85) | 132.332 (28.443-328.736) |
| Cameroon | 0.108 | 0.006 (0.001-0.015) | 0.532 (0.114-1.377) | 2.206 | 0.104 (0.022-0.271) | 11.069 (2.367-29.322) |
| Canada | 3.156 | 0.038 (0.008-0.092) | 27.609 (6.105-67.524) | 55.285 | 0.671 (0.15-1.638) | 470.104 (105.393-1148.292) |
| Central African Republic | 0.134 | 0.02 (0.004-0.054) | 0.336 (0.069-0.928) | 3.338 | 0.417 (0.085-1.136) | 8.529 (1.707-23.922) |
| Chad | 0.658 | 0.038 (0.008-0.093) | 1.732 (0.367-4.259) | 12.963 | 0.703 (0.15-1.734) | 35.578 (7.502-88.989) |
| Chile | 1.198 | 0.037 (0.009-0.088) | 8.993 (2.088-21.181) | 23.851 | 0.672 (0.157-1.606) | 163.382 (38.108-389.716) |
| China | 470.784 | 0.032 (0.008-0.073) | 629.447 (146.821-1423.356) | 11513.722 | 0.669 (0.156-1.499) | 13747.895 (3220.458-30821.154) |
| Colombia | 2.862 | 0.024 (0.006-0.059) | 12.605 (2.955-31.308) | 57.394 | 0.429 (0.1-1.083) | 224.527 (52.261-567.349) |
| Comoros | 0.037 | 0.025 (0.005-0.068) | 0.11 (0.022-0.293) | 0.738 | 0.465 (0.093-1.238) | 2.166 (0.43-5.711) |
| Congo | 0.162 | 0.022 (0.005-0.056) | 0.457 (0.093-1.197) | 3.799 | 0.433 (0.088-1.131) | 10.644 (2.199-28.23) |
| Cook Islands | 0.016 | 0.212 (0.052-0.499) | 0.053 (0.013-0.126) | 0.339 | 4.408 (1.088-10.339) | 1.11 (0.277-2.579) |
| Costa Rica | 0.644 | 0.058 (0.014-0.144) | 2.899 (0.676-7.24) | 12.019 | 1.07 (0.245-2.68) | 54.219 (12.545-135.789) |
| Croatia | 1.371 | 0.037 (0.008-0.093) | 3.523 (0.799-9.005) | 25.246 | 0.658 (0.154-1.647) | 60.016 (13.969-149.273) |
| Cuba | 5.784 | 0.031 (0.007-0.074) | 6.074 (1.427-14.738) | 104.654 | 0.538 (0.124-1.303) | 103.742 (24.144-250.719) |
| Cyprus | 0.181 | 0.029 (0.006-0.071) | 0.561 (0.125-1.376) | 3.224 | 0.467 (0.108-1.135) | 9.355 (2.138-22.889) |
| Czechia | 3.822 | 0.033 (0.008-0.079) | 7.557 (1.854-17.839) | 71.942 | 0.613 (0.151-1.444) | 133.367 (32.445-319.46) |
| Democratic People's Republic of Korea | 4.394 | 0.033 (0.007-0.086) | 10.259 (2.093-27.233) | 109.095 | 0.714 (0.146-1.925) | 233.908 (47.641-628.742) |
| Democratic Republic of the Congo | 1.16 | 0.012 (0.003-0.031) | 3.535 (0.758-9.389) | 27.662 | 0.245 (0.052-0.64) | 84.325 (17.065-216.379) |
| Denmark | 0.374 | 0.015 (0.003-0.039) | 1.879 (0.42-4.837) | 6.645 | 0.281 (0.062-0.706) | 32.911 (7.261-84.277) |
| Djibouti | 0.018 | 0.031 (0.006-0.085) | 0.134 (0.027-0.375) | 0.416 | 0.575 (0.115-1.606) | 3.015 (0.609-8.734) |
| Dominica | 0.063 | 0.051 (0.013-0.12) | 0.047 (0.012-0.11) | 1.096 | 0.945 (0.221-2.254) | 0.862 (0.202-2.052) |
| Dominican Republic | 0.445 | 0.033 (0.007-0.084) | 2.906 (0.614-7.292) | 8.889 | 0.612 (0.126-1.574) | 55.66 (11.435-144.216) |
| Ecuador | 1.072 | 0.056 (0.012-0.135) | 7.744 (1.704-18.595) | 18.663 | 0.88 (0.195-2.12) | 127.41 (27.806-307.151) |
| Egypt | 12.426 | 0.183 (0.039-0.456) | 107.165 (22.722-269.513) | 298.379 | 4.03 (0.853-10.183) | 2660.247 (535.859-6885.65) |
| El Salvador | 0.484 | 0.023 (0.005-0.057) | 1.417 (0.32-3.428) | 9.876 | 0.44 (0.102-1.088) | 25.861 (5.986-63.554) |
| Equatorial Guinea | 0.02 | 0.03 (0.006-0.079) | 0.113 (0.023-0.292) | 0.466 | 0.549 (0.11-1.449) | 2.405 (0.491-6.521) |
| Eritrea | 0.126 | 0.027 (0.005-0.07) | 0.529 (0.104-1.399) | 3.103 | 0.52 (0.102-1.367) | 12.345 (2.423-32.696) |
| Estonia | 0.148 | 0.02 (0.004-0.049) | 0.583 (0.121-1.475) | 2.873 | 0.369 (0.078-0.935) | 9.866 (2.057-24.603) |
| Eswatini | 0.1 | 0.19 (0.03-0.562) | 0.946 (0.141-2.823) | 2.133 | 3.841 (0.57-11.572) | 21.593 (2.993-66.771) |
| Ethiopia | 2.348 | 0.017 (0.004-0.04) | 5.709 (1.305-13.475) | 54.495 | 0.3 (0.068-0.711) | 112.049 (26.216-265.508) |
| Fiji | 0.196 | 0.13 (0.034-0.306) | 0.861 (0.218-1.993) | 4.801 | 2.754 (0.702-6.478) | 20.997 (5.202-48.907) |
| Finland | 1.045 | 0.031 (0.007-0.074) | 4.194 (0.968-10.305) | 18.232 | 0.522 (0.118-1.268) | 66.465 (15.265-160.997) |
| France | 8.096 | 0.021 (0.004-0.054) | 31.239 (6.39-81.766) | 147.395 | 0.382 (0.082-0.985) | 513.256 (108.861-1338.643) |
| Gabon | 0.075 | 0.029 (0.006-0.076) | 0.254 (0.054-0.65) | 1.62 | 0.56 (0.115-1.434) | 5.542 (1.113-14.288) |
| Gambia | 0.293 | 0.241 (0.051-0.598) | 2.045 (0.425-5.094) | 6.26 | 4.688 (0.993-11.884) | 42.925 (9.075-108.536) |
| Georgia | 0.257 | 0.025 (0.006-0.06) | 1.567 (0.357-3.758) | 5.067 | 0.527 (0.119-1.29) | 31.059 (6.959-75.578) |
| Germany | 22.042 | 0.042 (0.01-0.099) | 89.485 (20.605-214.822) | 373.743 | 0.741 (0.173-1.731) | 1445.376 (333.325-3423.53) |
| Ghana | 1.167 | 0.047 (0.01-0.12) | 6.092 (1.321-15.494) | 24.387 | 0.863 (0.188-2.207) | 125.979 (26.853-326.271) |
| Greece | 1.62 | 0.021 (0.005-0.053) | 6.144 (1.317-15.217) | 25.567 | 0.354 (0.077-0.896) | 88.871 (19.318-222.784) |
| Greenland | 0.004 | 0.051 (0.011-0.128) | 0.032 (0.007-0.083) | 0.103 | 0.998 (0.214-2.603) | 0.7 (0.149-1.867) |
| Grenada | 0.056 | 0.042 (0.011-0.1) | 0.043 (0.011-0.104) | 1 | 0.782 (0.193-1.901) | 0.865 (0.211-2.089) |
| Guam | 0.018 | 0.06 (0.014-0.145) | 0.112 (0.025-0.269) | 0.412 | 1.338 (0.296-3.228) | 2.576 (0.561-6.211) |
| Guatemala | 1.6 | 0.056 (0.013-0.136) | 5.755 (1.343-14.197) | 33.721 | 1.079 (0.248-2.693) | 117.145 (27.061-292.742) |
| Guinea | 3.033 | 0.199 (0.042-0.504) | 9.394 (1.996-24.035) | 59.6 | 3.682 (0.769-9.263) | 188.391 (39.604-479.182) |
| Guinea-Bissau | 0.107 | 0.047 (0.01-0.121) | 0.263 (0.055-0.678) | 2.352 | 0.898 (0.188-2.323) | 5.813 (1.215-15.184) |
| Guyana | 0.193 | 0.046 (0.011-0.109) | 0.253 (0.06-0.612) | 4.21 | 0.915 (0.214-2.216) | 5.619 (1.29-13.497) |
| Haiti | 1.219 | 0.043 (0.009-0.115) | 2.493 (0.502-6.77) | 27.219 | 0.833 (0.168-2.272) | 54.877 (11.289-150.133) |
| Honduras | 1.395 | 0.178 (0.03-0.502) | 9.763 (1.612-27.289) | 28.293 | 3.412 (0.56-9.656) | 200.593 (33.498-572.141) |
| Hungary | 5.302 | 0.023 (0.005-0.059) | 4.877 (1.085-12.322) | 98.791 | 0.42 (0.097-1.047) | 83.536 (19.311-207.907) |
| Iceland | 0.018 | 0.018 (0.004-0.044) | 0.103 (0.023-0.254) | 0.324 | 0.315 (0.07-0.769) | 1.74 (0.389-4.26) |
| India | 66.121 | 0.033 (0.008-0.075) | 342.275 (84.15-763.939) | 1524.326 | 0.646 (0.16-1.433) | 7226.342 (1795.659-16109.552) |
| Indonesia | 8.076 | 0.019 (0.004-0.043) | 32.633 (7.429-74.626) | 180.789 | 0.343 (0.078-0.779) | 680.602 (151.399-1530.261) |
| Iran (Islamic Republic of) | 7.039 | 0.062 (0.015-0.132) | 39.508 (9.607-84.418) | 150.049 | 1.114 (0.275-2.365) | 754.789 (186.55-1607.721) |
| Iraq | 2.802 | 0.094 (0.023-0.228) | 18.154 (4.242-44.325) | 61.453 | 1.865 (0.431-4.509) | 407.867 (93.683-987.348) |
| Ireland | 0.137 | 0.023 (0.005-0.056) | 1.782 (0.409-4.366) | 2.403 | 0.385 (0.086-0.956) | 29.526 (6.633-73.276) |
| Israel | 0.674 | 0.022 (0.005-0.055) | 2.71 (0.611-6.686) | 11.983 | 0.398 (0.09-0.993) | 46.413 (10.554-116.362) |
| Italy | 22.274 | 0.024 (0.006-0.053) | 39.18 (9.686-85.13) | 414.31 | 0.44 (0.106-0.957) | 630.413 (156.386-1378.888) |
| Jamaica | 0.542 | 0.038 (0.009-0.091) | 1.124 (0.272-2.706) | 9.912 | 0.705 (0.171-1.723) | 20.625 (4.937-50.298) |
| Japan | 47.339 | 0.027 (0.006-0.061) | 116.863 (26.983-265.637) | 1026.083 | 0.459 (0.104-1.034) | 1705.616 (388.558-3879.762) |
| Jordan | 0.35 | 0.044 (0.01-0.104) | 2.277 (0.524-5.528) | 7.483 | 0.804 (0.184-1.935) | 47.281 (10.983-112.834) |
| Kazakhstan | 1.481 | 0.052 (0.012-0.131) | 8.238 (1.791-20.441) | 32.86 | 1.056 (0.228-2.622) | 180.725 (37.83-443.615) |
| Kenya | 1.102 | 0.029 (0.006-0.075) | 5.104 (1.095-13.192) | 22.594 | 0.553 (0.118-1.433) | 113.119 (24.518-292.223) |
| Kiribati | 0.034 | 0.16 (0.037-0.373) | 0.098 (0.022-0.237) | 0.928 | 3.716 (0.84-8.929) | 2.761 (0.601-6.819) |
| Kuwait | 0.168 | 0.06 (0.015-0.144) | 1.13 (0.269-2.664) | 3.909 | 1.105 (0.267-2.63) | 23.761 (5.508-56.584) |
| Kyrgyzstan | 0.084 | 0.01 (0.002-0.026) | 0.389 (0.081-0.994) | 1.677 | 0.19 (0.039-0.495) | 8.138 (1.675-21.176) |
| Lao People's Democratic Republic | 0.851 | 0.065 (0.014-0.153) | 2.288 (0.494-5.563) | 19.43 | 1.213 (0.258-2.911) | 49.086 (10.483-119.346) |
| Latvia | 0.19 | 0.014 (0.003-0.037) | 0.638 (0.136-1.617) | 3.719 | 0.276 (0.06-0.711) | 11.09 (2.41-28.383) |
| Lebanon | 0.451 | 0.039 (0.009-0.092) | 2.025 (0.469-4.885) | 9.381 | 0.718 (0.166-1.73) | 37.568 (8.545-90.723) |
| Lesotho | 0.234 | 0.121 (0.024-0.338) | 1.334 (0.248-3.714) | 4.806 | 2.474 (0.454-6.959) | 30.557 (5.529-86.626) |
| Liberia | 0.371 | 0.054 (0.012-0.132) | 0.844 (0.183-2.088) | 7.365 | 0.957 (0.2-2.374) | 17.024 (3.524-42.981) |
| Libya | 0.829 | 0.102 (0.024-0.247) | 4.494 (1.042-11.116) | 17.678 | 2.061 (0.467-5.197) | 98.996 (22.265-253.102) |
| Lithuania | 0.218 | 0.012 (0.002-0.029) | 0.723 (0.146-1.867) | 4.221 | 0.226 (0.046-0.582) | 12.882 (2.631-33.074) |
| Luxembourg | 0.04 | 0.03 (0.007-0.075) | 0.317 (0.076-0.781) | 0.698 | 0.519 (0.123-1.297) | 5.2 (1.231-12.76) |
| Madagascar | 0.64 | 0.019 (0.004-0.05) | 1.54 (0.314-4.021) | 13.575 | 0.364 (0.074-0.95) | 35.665 (7.211-94.749) |
| Malawi | 0.673 | 0.03 (0.006-0.074) | 1.765 (0.382-4.289) | 14.74 | 0.547 (0.118-1.35) | 35.778 (7.792-87.633) |
| Malaysia | 3.342 | 0.088 (0.02-0.211) | 20.249 (4.616-48.834) | 68.498 | 1.576 (0.361-3.779) | 400.386 (90.088-962.448) |
| Maldives | 0.023 | 0.06 (0.014-0.149) | 0.144 (0.032-0.36) | 0.49 | 0.975 (0.217-2.386) | 2.55 (0.56-6.329) |
| Mali | 1.41 | 0.069 (0.015-0.178) | 5.198 (1.077-13.267) | 32.406 | 1.417 (0.289-3.672) | 117.642 (24.049-309.16) |
| Malta | 0.04 | 0.019 (0.004-0.045) | 0.189 (0.045-0.457) | 0.738 | 0.332 (0.079-0.808) | 3.204 (0.78-7.768) |
| Marshall Islands | 0.017 | 0.183 (0.045-0.435) | 0.053 (0.013-0.128) | 0.422 | 4.058 (1.003-9.71) | 1.478 (0.366-3.61) |
| Mauritania | 0.249 | 0.034 (0.007-0.086) | 0.606 (0.12-1.548) | 4.939 | 0.595 (0.117-1.521) | 11.449 (2.247-29.998) |
| Mauritius | 0.112 | 0.042 (0.011-0.098) | 0.69 (0.174-1.598) | 2.338 | 0.766 (0.192-1.784) | 13.216 (3.192-31.07) |
| Mexico | 10.536 | 0.056 (0.014-0.117) | 61.711 (15.909-129.43) | 205.341 | 1.06 (0.27-2.248) | 1219.91 (308.184-2594.518) |
| Micronesia (Federated States of) | 0.031 | 0.158 (0.038-0.375) | 0.095 (0.022-0.233) | 0.734 | 3.417 (0.784-8.471) | 2.471 (0.57-6.299) |
| Monaco | 0.009 | 0.053 (0.011-0.131) | 0.056 (0.012-0.14) | 0.147 | 0.948 (0.208-2.474) | 0.911 (0.196-2.345) |
| Mongolia | 0.846 | 0.353 (0.068-0.917) | 5.754 (1.123-15.041) | 19.177 | 6.175 (1.188-16.219) | 125.771 (25.231-326.415) |
| Montenegro | 0.209 | 0.06 (0.013-0.14) | 0.601 (0.128-1.411) | 4.095 | 1.129 (0.248-2.699) | 11.431 (2.54-27.652) |
| Morocco | 1.534 | 0.035 (0.008-0.085) | 9.126 (2.022-22.202) | 29.95 | 0.62 (0.136-1.513) | 178.537 (39.64-442.427) |
| Mozambique | 0.582 | 0.033 (0.007-0.082) | 2.759 (0.576-6.837) | 11.703 | 0.594 (0.127-1.465) | 57.328 (11.783-144.316) |
| Myanmar | 3.65 | 0.053 (0.012-0.127) | 20.63 (4.531-49.695) | 76.169 | 0.955 (0.21-2.304) | 412.793 (87.942-995.737) |
| Namibia | 0.08 | 0.028 (0.006-0.068) | 0.345 (0.073-0.854) | 1.657 | 0.53 (0.112-1.339) | 7.117 (1.479-17.875) |
| Nauru | 0.002 | 0.127 (0.03-0.319) | 0.004 (0.001-0.01) | 0.058 | 2.744 (0.634-6.862) | 0.119 (0.026-0.319) |
| Nepal | 0.594 | 0.021 (0.004-0.054) | 4.044 (0.858-10.074) | 12.913 | 0.376 (0.078-0.955) | 79.277 (16.275-205.41) |
| Netherlands | 1.223 | 0.016 (0.003-0.04) | 6.03 (1.298-14.767) | 20.708 | 0.281 (0.062-0.677) | 98.343 (21.947-235.901) |
| New Zealand | 0.29 | 0.028 (0.006-0.062) | 2.284 (0.525-5.092) | 5.714 | 0.524 (0.12-1.164) | 40.615 (9.27-90.151) |
| Nicaragua | 0.263 | 0.043 (0.01-0.102) | 1.684 (0.393-4.084) | 5.549 | 0.807 (0.186-1.961) | 34.197 (7.771-83.073) |
| Niger | 0.028 | 0.003 (0.001-0.007) | 0.175 (0.037-0.462) | 0.603 | 0.053 (0.011-0.14) | 3.704 (0.776-9.991) |
| Nigeria | 5.747 | 0.024 (0.005-0.055) | 16.032 (3.481-36.468) | 110.465 | 0.419 (0.089-0.964) | 310.161 (66.679-722.16) |
| Niue | 0.002 | 0.15 (0.039-0.34) | 0.003 (0.001-0.007) | 0.039 | 3.23 (0.829-7.624) | 0.071 (0.018-0.168) |
| North Macedonia | 0.908 | 0.104 (0.025-0.253) | 3.241 (0.764-8.057) | 18.767 | 1.93 (0.443-4.714) | 63.885 (14.83-156.849) |
| Northern Mariana Islands | 0.008 | 0.108 (0.025-0.255) | 0.05 (0.011-0.122) | 0.216 | 2.261 (0.506-5.501) | 1.26 (0.265-3.116) |
| Norway | 0.505 | 0.016 (0.004-0.035) | 1.634 (0.384-3.613) | 8.661 | 0.285 (0.067-0.623) | 27.347 (6.539-60.21) |
| Oman | 0.142 | 0.08 (0.019-0.191) | 1.002 (0.225-2.438) | 3.396 | 1.558 (0.357-3.756) | 23.967 (5.479-58.372) |
| Pakistan | 7.381 | 0.026 (0.005-0.062) | 24.658 (5.136-57.7) | 164.946 | 0.541 (0.113-1.264) | 591.235 (125.468-1395.348) |
| Palau | 0.006 | 0.138 (0.032-0.323) | 0.027 (0.006-0.066) | 0.143 | 3.125 (0.694-7.719) | 0.72 (0.151-1.788) |
| Palestine | 0.562 | 0.105 (0.024-0.248) | 1.935 (0.444-4.619) | 11.141 | 1.906 (0.439-4.604) | 40.011 (8.899-97.959) |
| Panama | 0.279 | 0.031 (0.007-0.075) | 1.281 (0.3-3.076) | 5.41 | 0.582 (0.131-1.411) | 23.875 (5.365-57.685) |
| Papua New Guinea | 0.122 | 0.017 (0.004-0.041) | 0.559 (0.126-1.353) | 2.712 | 0.309 (0.069-0.75) | 12.433 (2.778-30.58) |
| Paraguay | 0.157 | 0.012 (0.003-0.03) | 0.616 (0.131-1.556) | 3.046 | 0.219 (0.046-0.567) | 11.692 (2.429-30.215) |
| Peru | 2.481 | 0.02 (0.004-0.051) | 6.471 (1.335-16.001) | 45.207 | 0.338 (0.067-0.854) | 105.874 (20.738-265.606) |
| Philippines | 11.612 | 0.044 (0.01-0.098) | 28.897 (6.763-65.145) | 242.293 | 0.842 (0.197-1.887) | 627.372 (143.493-1429.806) |
| Poland | 20.349 | 0.017 (0.004-0.038) | 12.803 (3.059-28.055) | 366.798 | 0.306 (0.074-0.674) | 219.49 (52.635-484.736) |
| Portugal | 1.221 | 0.033 (0.008-0.079) | 8.667 (1.946-20.627) | 22.537 | 0.631 (0.145-1.538) | 148.039 (33.654-354.839) |
| Puerto Rico | 3.161 | 0.054 (0.014-0.129) | 4.208 (1.074-9.918) | 56.3 | 1.005 (0.252-2.409) | 72.081 (18.161-170.177) |
| Qatar | 0.219 | 0.63 (0.165-1.413) | 2.61 (0.614-6.059) | 4.984 | 10.429 (2.598-23.367) | 63.819 (14.676-153.018) |
| Republic of Korea | 8.89 | 0.085 (0.019-0.202) | 75.628 (16.856-181.136) | 199.188 | 1.497 (0.338-3.688) | 1362.828 (307.434-3356.685) |
| Republic of Moldova | 0.191 | 0.012 (0.002-0.029) | 0.686 (0.143-1.689) | 4.104 | 0.22 (0.046-0.554) | 12.872 (2.685-32.538) |
| Romania | 1.578 | 0.017 (0.004-0.041) | 6.561 (1.406-16.358) | 31.004 | 0.325 (0.068-0.818) | 121.247 (25.524-303.137) |
| Russian Federation | 8.162 | 0.013 (0.003-0.03) | 31.473 (7.103-72.197) | 169.117 | 0.251 (0.057-0.576) | 595.631 (137.1-1370.649) |
| Rwanda | 0.619 | 0.033 (0.007-0.083) | 1.569 (0.332-3.936) | 13.699 | 0.615 (0.129-1.538) | 33.736 (6.948-86.481) |
| Saint Helena | 0.871 | 0.042 (0.009-0.102) | 3.184 (0.675-7.931) | 19.147 | 0.736 (0.154-1.846) | 66.307 (13.52-167.39) |
| Saint Kitts and Nevis | 0.053 | 0.056 (0.014-0.128) | 0.031 (0.007-0.072) | 0.968 | 1.045 (0.246-2.437) | 0.662 (0.153-1.585) |
| Saint Lucia | 0.059 | 0.035 (0.009-0.082) | 0.074 (0.018-0.17) | 1.118 | 0.644 (0.157-1.506) | 1.373 (0.335-3.204) |
| Saint Vincent and the Grenadines | 0.052 | 0.043 (0.011-0.101) | 0.057 (0.014-0.133) | 0.952 | 0.821 (0.198-1.918) | 1.115 (0.271-2.614) |
| Samoa | 0.039 | 0.065 (0.015-0.153) | 0.089 (0.02-0.209) | 0.892 | 1.433 (0.326-3.408) | 2.112 (0.481-5.066) |
| San Marino | 0.003 | 0.018 (0.004-0.049) | 0.012 (0.003-0.033) | 0.045 | 0.31 (0.064-0.859) | 0.199 (0.042-0.556) |
| Sao Tome and Principe | 0.008 | 0.025 (0.005-0.062) | 0.021 (0.004-0.052) | 0.148 | 0.439 (0.096-1.109) | 0.409 (0.089-1.044) |
| Saudi Arabia | 2.971 | 0.12 (0.029-0.286) | 14.081 (3.336-33.969) | 63.36 | 2.283 (0.543-5.484) | 330.587 (76.407-803.678) |
| Senegal | 0.532 | 0.031 (0.006-0.076) | 1.935 (0.41-4.73) | 10.452 | 0.554 (0.115-1.357) | 38.149 (7.656-95.135) |
| Serbia | 3.103 | 0.047 (0.011-0.112) | 7.909 (1.841-19.055) | 64.651 | 0.859 (0.201-2.117) | 144.013 (33.59-346.961) |
| Seychelles | 0.034 | 0.102 (0.025-0.236) | 0.1 (0.025-0.226) | 0.639 | 1.903 (0.468-4.451) | 2.02 (0.492-4.719) |
| Sierra Leone | 0.251 | 0.026 (0.005-0.067) | 0.762 (0.154-1.94) | 4.67 | 0.462 (0.092-1.197) | 15.082 (2.981-39.019) |
| Singapore | 0.609 | 0.044 (0.01-0.108) | 3.183 (0.709-7.828) | 12.662 | 0.736 (0.166-1.843) | 55.823 (12.424-141.155) |
| Slovakia | 1.138 | 0.022 (0.005-0.058) | 2.107 (0.459-5.499) | 22.325 | 0.416 (0.09-1.081) | 39.349 (8.627-102.539) |
| Slovenia | 0.405 | 0.041 (0.009-0.106) | 1.911 (0.397-4.928) | 7.405 | 0.746 (0.158-1.964) | 32.667 (6.926-85.502) |
| Solomon Islands | 0.039 | 0.06 (0.015-0.139) | 0.155 (0.035-0.376) | 1.124 | 1.334 (0.304-3.207) | 4.35 (0.961-10.664) |
| Somalia | 0.38 | 0.028 (0.005-0.076) | 1.377 (0.27-3.917) | 8.854 | 0.54 (0.106-1.514) | 32.087 (6.05-89.021) |
| South Africa | 8.099 | 0.072 (0.017-0.154) | 28.594 (6.841-61.5) | 158.664 | 1.361 (0.33-2.944) | 590.556 (137.795-1267.987) |
| South Sudan | 0.379 | 0.025 (0.005-0.067) | 0.742 (0.143-2.029) | 7.673 | 0.461 (0.088-1.257) | 15.708 (2.968-44.455) |
| Spain | 10.069 | 0.032 (0.007-0.078) | 33.865 (7.515-82.571) | 179.629 | 0.576 (0.129-1.46) | 545.084 (122.687-1349.989) |
| Sri Lanka | 1.644 | 0.048 (0.012-0.115) | 11.335 (2.674-27.162) | 31.085 | 0.851 (0.204-2.031) | 215.979 (51.227-523.308) |
| Sudan | 2.237 | 0.07 (0.015-0.179) | 10.691 (2.183-27.48) | 45.674 | 1.287 (0.253-3.341) | 215.94 (42.659-563.105) |
| Suriname | 0.126 | 0.041 (0.01-0.096) | 0.229 (0.056-0.538) | 2.573 | 0.781 (0.181-1.875) | 4.639 (1.058-11.192) |
| Sweden | 1.832 | 0.02 (0.005-0.044) | 4.569 (1.093-10.207) | 30.497 | 0.36 (0.085-0.801) | 75.477 (18.128-168.846) |
| Switzerland | 0.934 | 0.024 (0.005-0.059) | 4.468 (0.98-11.074) | 16.159 | 0.428 (0.096-1.1) | 74.445 (16.771-189.102) |
| Syrian Arab Republic | 1.821 | 0.068 (0.015-0.168) | 6.895 (1.54-17.383) | 37.403 | 1.23 (0.276-3.094) | 143.233 (32.867-366.535) |
| Taiwan (Province of China) | 5.758 | 0.053 (0.012-0.138) | 21.181 (4.664-54.957) | 137.722 | 1.002 (0.221-2.656) | 399.782 (87.939-1068.541) |
| Tajikistan | 0.054 | 0.021 (0.004-0.052) | 0.716 (0.153-1.869) | 1.117 | 0.38 (0.081-0.976) | 16.725 (3.541-44.442) |
| Thailand | 28.425 | 0.218 (0.05-0.546) | 216.08 (50.052-535.488) | 606.932 | 4.014 (0.904-9.91) | 4090.074 (906.615-10094.439) |
| Timor-Leste | 0.06 | 0.055 (0.012-0.134) | 0.389 (0.08-0.961) | 1.386 | 1.004 (0.207-2.534) | 7.895 (1.602-20.522) |
| Togo | 0.203 | 0.031 (0.006-0.078) | 0.847 (0.172-2.122) | 4.187 | 0.561 (0.115-1.41) | 17.842 (3.573-44.863) |
| Tokelau | 0.001 | 0.107 (0.025-0.257) | 0.001 (0-0.003) | 0.019 | 2.233 (0.522-5.487) | 0.03 (0.007-0.074) |
| Tonga | 0.092 | 0.337 (0.079-0.813) | 0.261 (0.062-0.63) | 2.154 | 7.436 (1.723-17.916) | 5.892 (1.357-14.147) |
| Trinidad and Tobago | 0.705 | 0.048 (0.013-0.115) | 0.863 (0.222-2.098) | 14.275 | 0.91 (0.225-2.24) | 17.042 (4.133-41.876) |
| Tunisia | 0.463 | 0.02 (0.005-0.048) | 2.352 (0.541-5.714) | 9.418 | 0.376 (0.084-0.922) | 46.709 (10.333-115.402) |
| Turkey | 7.131 | 0.035 (0.008-0.084) | 28.544 (6.247-69.165) | 146.371 | 0.604 (0.127-1.479) | 515.075 (109.632-1264.769) |
| Turkmenistan | 0.052 | 0.028 (0.006-0.072) | 1.035 (0.218-2.703) | 1.083 | 0.652 (0.137-1.703) | 26.622 (5.498-68.299) |
| Tuvalu | 0.004 | 0.114 (0.028-0.273) | 0.011 (0.003-0.026) | 0.105 | 2.433 (0.571-5.866) | 0.252 (0.058-0.615) |
| Uganda | 1.435 | 0.055 (0.011-0.139) | 6.393 (1.309-15.861) | 30.419 | 1.071 (0.221-2.711) | 140.573 (28.997-363.194) |
| Ukraine | 2.4 | 0.011 (0.002-0.025) | 8.854 (1.972-20.17) | 45.151 | 0.216 (0.048-0.49) | 166.292 (37.319-375.278) |
| United Arab Emirates | 0.145 | 0.117 (0.02-0.435) | 2.428 (0.385-9.161) | 3.537 | 2.226 (0.376-8.241) | 70.775 (10.909-261.587) |
| United Kingdom | 8.51 | 0.037 (0.009-0.079) | 51.713 (12.886-109.253) | 147.337 | 0.649 (0.164-1.391) | 828.631 (210.504-1754.759) |
| United Republic of Tanzania | 0.81 | 0.019 (0.004-0.048) | 3.888 (0.832-9.595) | 16.171 | 0.348 (0.075-0.848) | 77.307 (15.911-188.646) |
| United States of America | 57.976 | 0.058 (0.015-0.124) | 336.384 (85.951-714.09) | 1082.062 | 1.149 (0.293-2.462) | 6444.538 (1647.517-13780.423) |
| United States Virgin Islands | 0.029 | 0.043 (0.01-0.102) | 0.082 (0.019-0.193) | 0.583 | 0.808 (0.187-1.947) | 1.571 (0.371-3.779) |
| Uruguay | 0.142 | 0.017 (0.004-0.042) | 1.018 (0.226-2.481) | 2.534 | 0.317 (0.07-0.78) | 17.345 (3.829-42.696) |
| Uzbekistan | 0.217 | 0.045 (0.01-0.11) | 7.339 (1.551-17.843) | 4.33 | 0.92 (0.198-2.24) | 186.59 (38.62-457) |
| Vanuatu | 0.031 | 0.104 (0.024-0.265) | 0.161 (0.036-0.407) | 0.739 | 2.232 (0.484-5.773) | 3.906 (0.852-10.255) |
| Venezuela (Bolivarian Republic of) | 3.84 | 0.025 (0.006-0.063) | 6.884 (1.639-17.346) | 77.952 | 0.469 (0.11-1.199) | 133.564 (30.951-340.061) |
| Viet Nam | 7.272 | 0.028 (0.006-0.067) | 21.036 (4.804-51.078) | 134.741 | 0.454 (0.104-1.115) | 371.105 (82.688-912.176) |
| Yemen | 0.541 | 0.026 (0.006-0.069) | 2.812 (0.603-7.478) | 11.856 | 0.492 (0.103-1.316) | 59.593 (12.495-161.758) |
| Zambia | 0.371 | 0.028 (0.006-0.069) | 1.432 (0.306-3.529) | 7.722 | 0.509 (0.109-1.244) | 29.831 (6.39-73.157) |
| Zimbabwe | 2.284 | 0.131 (0.028-0.323) | 7.744 (1.686-19.464) | 49.106 | 2.643 (0.57-6.716) | 177.156 (37.373-453.198) |

NASH: nonalcoholic steatohepatitis; HFPG: high fasting plasma glucose; DALYs: disability-adjusted life years; ASR: age standardized rate.

Table S5 EAPC of NASH-related liver cancer deaths and DALYs attributable to HPFG in countries and territories.

| location | EAPC (deaths) | EAPC (DALYs) |
| --- | --- | --- |
| Afghanistan | 2.15 (2.11-2.19) | 2.13 (2.08-2.18) |
| Albania | -1.59 (-2.21--0.96) | -1.5 (-2.11--0.88) |
| Algeria | 3.63 (3.51-3.75) | 3.65 (3.54-3.77) |
| American Samoa | 2.37 (1.9-2.84) | 2.55 (2.06-3.05) |
| Andorra | 2.35 (2.24-2.47) | 2.41 (2.31-2.51) |
| Angola | 1.22 (1.16-1.28) | 1.16 (1.1-1.22) |
| Antigua and Barbuda | -1.98 (-2.91--1.05) | -2.13 (-3.06--1.2) |
| Argentina | 3.97 (3.77-4.17) | 3.96 (3.78-4.14) |
| Armenia | 12.77 (11.03-14.54) | 12.71 (10.98-14.46) |
| Australia | 5.75 (5.19-6.31) | 5.73 (5.23-6.23) |
| Austria | 4.13 (3.7-4.55) | 4.05 (3.63-4.47) |
| Azerbaijan | 9.82 (7.71-11.97) | 9.65 (7.43-11.91) |
| Bahamas | -1.77 (-2.57--0.96) | -1.81 (-2.62--0.98) |
| Bahrain | 1.26 (0.73-1.79) | 0.94 (0.38-1.5) |
| Bangladesh | 1.33 (1.03-1.63) | 0.97 (0.78-1.16) |
| Barbados | 1.07 (0.91-1.22) | 1.07 (0.89-1.26) |
| Belarus | 3.44 (2.9-3.99) | 3.62 (2.99-4.26) |
| Belgium | 2.1 (1.67-2.54) | 2.41 (2.02-2.8) |
| Belize | -0.71 (-1.13--0.28) | -0.62 (-1.02--0.22) |
| Benin | 1.32 (1.16-1.49) | 1.26 (1.07-1.45) |
| Bermuda | -3 (-4.02--1.97) | -3.11 (-4.15--2.06) |
| Bhutan | 4.47 (4.31-4.64) | 4.11 (3.97-4.26) |
| Bolivia (Plurinational State of) | 1.73 (1.65-1.82) | 1.55 (1.46-1.64) |
| Bosnia and Herzegovina | 5.53 (4.88-6.18) | 5.19 (4.59-5.8) |
| Botswana | 2.93 (2.29-3.57) | 3.08 (2.39-3.77) |
| Brazil | 2.27 (1.94-2.61) | 2.2 (1.88-2.51) |
| Brunei Darussalam | 1.62 (0.99-2.24) | 1.7 (1.09-2.32) |
| Bulgaria | 0.65 (0.04-1.26) | 0.67 (0.06-1.29) |
| Burkina Faso | 0.73 (0.57-0.89) | 0.77 (0.58-0.97) |
| Burundi | -0.13 (-0.33-0.06) | -0.23 (-0.43--0.03) |
| Cabo Verde | 9.61 (7.29-11.99) | 9.63 (7.28-12.04) |
| Cambodia | 2.84 (2.65-3.05) | 2.62 (2.4-2.84) |
| Cameroon | 1.71 (1.08-2.35) | 1.85 (1.32-2.39) |
| Canada | 4.96 (4.74-5.19) | 5.02 (4.8-5.24) |
| Central African Republic | 0.72 (0.45-0.99) | 0.69 (0.42-0.96) |
| Chad | 1.23 (1.03-1.44) | 1.2 (1-1.41) |
| Chile | 4.53 (4.27-4.79) | 4.32 (4.07-4.56) |
| China | -3.52 (-4.17--2.86) | -3.84 (-4.55--3.13) |
| Colombia | 0.81 (0.44-1.18) | 0.66 (0.29-1.03) |
| Comoros | 0.82 (0.71-0.93) | 0.84 (0.71-0.97) |
| Congo | 0.27 (0.11-0.44) | 0.14 (-0.04-0.32) |
| Cook Islands | 1.38 (1.13-1.63) | 1.53 (1.32-1.74) |
| Costa Rica | 0.95 (0.37-1.54) | 1.02 (0.41-1.63) |
| Croatia | 1.93 (1.38-2.48) | 2.01 (1.44-2.57) |
| Cuba | -2.34 (-3.71--0.95) | -2.53 (-3.9--1.13) |
| Cyprus | 0.53 (0.29-0.78) | 0.56 (0.37-0.74) |
| Czechia | 0.42 (0.25-0.59) | 0.38 (0.21-0.54) |
| Democratic People's Republic of Korea | -0.08 (-0.18-0.02) | -0.05 (-0.18-0.07) |
| Democratic Republic of the Congo | 0.59 (0.5-0.67) | 0.72 (0.62-0.82) |
| Denmark | 4.89 (4.56-5.22) | 4.83 (4.5-5.16) |
| Djibouti | 1.65 (1.5-1.8) | 1.65 (1.5-1.8) |
| Dominica | -1.71 (-2.38--1.04) | -1.71 (-2.41--1.01) |
| Dominican Republic | 3.75 (3.12-4.39) | 3.68 (3.03-4.33) |
| Ecuador | 3.72 (3.4-4.05) | 3.42 (3.13-3.7) |
| Egypt | 5.68 (5.24-6.13) | 5.81 (5.36-6.26) |
| El Salvador | 0.88 (0.37-1.39) | 0.69 (0.12-1.27) |
| Equatorial Guinea | 3.55 (3.41-3.69) | 3.15 (2.99-3.31) |
| Eritrea | 1.2 (0.97-1.43) | 1.12 (0.91-1.33) |
| Estonia | 3.01 (2.34-3.68) | 2.68 (2-3.37) |
| Eswatini | 6.15 (4.75-7.56) | 6.52 (5.03-8.03) |
| Ethiopia | -0.03 (-0.19-0.13) | -0.45 (-0.64--0.27) |
| Fiji | 2.44 (2.33-2.55) | 2.65 (2.5-2.79) |
| Finland | 3.08 (2.89-3.27) | 3.02 (2.81-3.22) |
| France | 2.58 (2.25-2.91) | 2.34 (2.03-2.65) |
| Gabon | 2.04 (1.73-2.35) | 2.01 (1.69-2.32) |
| Gambia | 2.76 (2.53-2.99) | 2.86 (2.61-3.11) |
| Georgia | 5.52 (4.49-6.55) | 5.92 (4.76-7.09) |
| Germany | 3.5 (3.15-3.84) | 3.56 (3.33-3.79) |
| Ghana | 2.47 (2.02-2.93) | 2.51 (2.08-2.95) |
| Greece | 2.76 (2.58-2.95) | 3.05 (2.9-3.21) |
| Greenland | 4.17 (4.03-4.3) | 3.99 (3.89-4.1) |
| Grenada | -2.05 (-2.96--1.13) | -2.15 (-3.11--1.18) |
| Guam | 2.66 (2.41-2.9) | 3.38 (3.15-3.61) |
| Guatemala | -0.91 (-2.22-0.42) | -0.65 (-2.02-0.75) |
| Guinea | 2.15 (1.96-2.34) | 2.28 (2.13-2.42) |
| Guinea-Bissau | 1.16 (1.06-1.26) | 1.09 (1-1.19) |
| Guyana | -0.96 (-1.7--0.22) | -1 (-1.72--0.28) |
| Haiti | -0.25 (-0.48--0.03) | -0.25 (-0.49--0.01) |
| Honduras | 3.35 (3.14-3.57) | 3.36 (3.16-3.55) |
| Hungary | -0.89 (-1.76-0) | -0.88 (-1.82-0.07) |
| Iceland | 3.68 (3.48-3.89) | 3.58 (3.4-3.76) |
| India | 1.62 (1.48-1.76) | 1.69 (1.58-1.81) |
| Indonesia | 2.19 (2.04-2.34) | 1.85 (1.77-1.93) |
| Iran (Islamic Republic of) | 1.6 (1-2.19) | 1.61 (1.01-2.22) |
| Iraq | 3.25 (2.78-3.72) | 3.13 (2.66-3.59) |
| Ireland | 7.97 (7.6-8.35) | 7.81 (7.45-8.17) |
| Israel | 1.9 (1.43-2.37) | 1.72 (1.33-2.12) |
| Italy | -0.1 (-0.42-0.21) | -0.35 (-0.62--0.07) |
| Jamaica | 1.6 (0.87-2.34) | 1.57 (0.8-2.35) |
| Japan | -1.19 (-1.78--0.59) | -2.04 (-2.65--1.44) |
| Jordan | 0.58 (0.36-0.8) | 0.45 (0.22-0.69) |
| Kazakhstan | 2.46 (1.53-3.4) | 2.06 (1.05-3.08) |
| Kenya | 1.44 (1.06-1.82) | 1.61 (1.23-2) |
| Kiribati | 1.48 (1.22-1.73) | 1.41 (1.16-1.67) |
| Kuwait | 2.55 (2.18-2.93) | 2.29 (1.91-2.68) |
| Kyrgyzstan | 5.02 (4.71-5.33) | 4.95 (4.67-5.22) |
| Lao People's Democratic Republic | 0.7 (0.58-0.82) | 0.45 (0.32-0.59) |
| Latvia | 3.12 (2.3-3.94) | 2.88 (2.04-3.73) |
| Lebanon | 1.82 (1.7-1.93) | 1.77 (1.63-1.92) |
| Lesotho | 5.92 (5.29-6.55) | 6.32 (5.66-6.98) |
| Liberia | 1.34 (1.12-1.56) | 1.31 (1.05-1.56) |
| Libya | 2.61 (2.45-2.76) | 2.69 (2.54-2.84) |
| Lithuania | 2.58 (2.18-2.98) | 2.5 (2.08-2.92) |
| Luxembourg | 5.51 (5.23-5.78) | 5.44 (5.15-5.72) |
| Madagascar | 0.66 (0.55-0.78) | 0.7 (0.58-0.82) |
| Malawi | 0.52 (0.23-0.82) | 0.27 (-0.05-0.6) |
| Malaysia | 2.75 (2.28-3.22) | 2.64 (2.23-3.05) |
| Maldives | 1.72 (1.54-1.89) | 1.44 (1.22-1.65) |
| Mali | 1.92 (1.84-1.99) | 1.87 (1.79-1.95) |
| Malta | 2.38 (1.97-2.8) | 2.34 (1.93-2.74) |
| Marshall Islands | 1.28 (1.11-1.45) | 1.4 (1.21-1.59) |
| Mauritania | 0.16 (-0.01-0.33) | 0.08 (-0.08-0.23) |
| Mauritius | 4.73 (4.28-5.19) | 4.65 (4.14-5.16) |
| Mexico | 2.39 (2.23-2.54) | 2.64 (2.49-2.8) |
| Micronesia (Federated States of) | 2.38 (2.01-2.76) | 2.48 (2.07-2.89) |
| Monaco | 6.24 (5.32-7.17) | 6.22 (5.32-7.13) |
| Mongolia | 6.38 (5.61-7.16) | 5.45 (4.79-6.11) |
| Montenegro | 1.73 (1.53-1.94) | 1.68 (1.44-1.92) |
| Morocco | 3.24 (2.98-3.51) | 3.29 (3.05-3.52) |
| Mozambique | 3.16 (2.95-3.36) | 3.38 (3.17-3.59) |
| Myanmar | 3.58 (3.3-3.86) | 3.48 (3.23-3.73) |
| Namibia | 2.7 (2.26-3.14) | 2.78 (2.33-3.23) |
| Nauru | 1.28 (1.01-1.54) | 1.44 (1.17-1.71) |
| Nepal | 3.27 (3.1-3.43) | 3.11 (2.92-3.31) |
| Netherlands | 3.88 (3.73-4.03) | 3.84 (3.69-3.98) |
| New Zealand | 4.38 (4.17-4.6) | 3.97 (3.7-4.24) |
| Nicaragua | 2.9 (2.62-3.18) | 2.76 (2.38-3.13) |
| Niger | 2.38 (2.26-2.5) | 2.42 (2.24-2.6) |
| Nigeria | 1.07 (0.89-1.24) | 1.06 (0.85-1.26) |
| Niue | 1.8 (1.58-2.02) | 1.94 (1.71-2.17) |
| North Macedonia | 2.43 (2.23-2.62) | 2.23 (2.03-2.43) |
| Northern Mariana Islands | 1.96 (1.8-2.12) | 2.06 (1.89-2.22) |
| Norway | 3.12 (3.02-3.21) | 2.97 (2.88-3.07) |
| Oman | 4.81 (4.41-5.21) | 4.51 (4.1-4.92) |
| Pakistan | 2.47 (2.28-2.67) | 2.41 (2.21-2.6) |
| Palau | 2.36 (2.04-2.67) | 2.57 (2.24-2.9) |
| Palestine | 1.28 (1.2-1.36) | 1.25 (1.15-1.35) |
| Panama | 1.82 (1.44-2.21) | 1.81 (1.39-2.22) |
| Papua New Guinea | 2.17 (2.07-2.28) | 2.18 (2.06-2.29) |
| Paraguay | 1.53 (0.96-2.11) | 1.44 (0.84-2.05) |
| Peru | -1.41 (-2--0.82) | -1.68 (-2.32--1.04) |
| Philippines | -0.9 (-1.2--0.59) | -0.71 (-1--0.41) |
| Poland | -3.34 (-4.89--1.76) | -3.24 (-4.78--1.67) |
| Portugal | 4.47 (3.97-4.97) | 4.66 (4.15-5.17) |
| Puerto Rico | -0.47 (-1.21-0.26) | -0.31 (-0.99-0.39) |
| Qatar | 2.05 (1.77-2.33) | 1.64 (1.3-1.97) |
| Republic of Korea | 3.32 (2.11-4.54) | 3.01 (1.71-4.32) |
| Republic of Moldova | 1.82 (0.43-3.24) | 1.27 (-0.31-2.87) |
| Romania | 4.24 (3.79-4.69) | 4.55 (4.06-5.04) |
| Russian Federation | 4.31 (4-4.62) | 4.14 (3.81-4.46) |
| Rwanda | 0.24 (-0.02-0.5) | 0.04 (-0.25-0.33) |
| Saint Helena | 0.28 (-0.01-0.56) | 0.2 (-0.1-0.49) |
| Saint Kitts and Nevis | -3.08 (-4.04--2.11) | -3.19 (-4.17--2.2) |
| Saint Lucia | -2.89 (-3.92--1.85) | -2.83 (-3.89--1.76) |
| Saint Vincent and the Grenadines | -1.66 (-2.58--0.73) | -1.55 (-2.44--0.66) |
| Samoa | 0.79 (0.64-0.95) | 0.89 (0.71-1.06) |
| San Marino | 3.52 (3.28-3.76) | 3.62 (3.37-3.86) |
| Sao Tome and Principe | 2.05 (2.01-2.09) | 2.08 (2.03-2.14) |
| Saudi Arabia | 1.96 (1.57-2.36) | 1.95 (1.57-2.33) |
| Senegal | 1.86 (1.65-2.08) | 1.95 (1.72-2.18) |
| Serbia | 1.37 (1.11-1.64) | 1.11 (0.82-1.4) |
| Seychelles | 2.07 (1.94-2.21) | 2 (1.87-2.14) |
| Sierra Leone | 2.09 (2.01-2.17) | 2.21 (2.14-2.29) |
| Singapore | 1.27 (0.84-1.71) | 0.71 (0.27-1.16) |
| Slovakia | -0.2 (-0.56-0.17) | -0.31 (-0.67-0.04) |
| Slovenia | 3.25 (2.82-3.69) | 3.27 (2.81-3.73) |
| Solomon Islands | 2.18 (1.95-2.4) | 1.99 (1.79-2.19) |
| Somalia | 1.14 (1.02-1.27) | 1.16 (1.03-1.28) |
| South Africa | 1.3 (0.64-1.97) | 1.54 (0.88-2.21) |
| South Sudan | 1.04 (0.96-1.13) | 1.08 (1.01-1.16) |
| Spain | 1.67 (1.1-2.25) | 1.56 (0.99-2.14) |
| Sri Lanka | 4.39 (4.01-4.77) | 4.49 (4.11-4.87) |
| Sudan | 3.27 (3.07-3.46) | 3.23 (3.03-3.43) |
| Suriname | -0.69 (-1.51-0.13) | -0.74 (-1.56-0.09) |
| Sweden | 2.71 (2.21-3.21) | 2.83 (2.32-3.34) |
| Switzerland | 3.32 (2.74-3.9) | 3.21 (2.63-3.8) |
| Syrian Arab Republic | 1.35 (1.19-1.52) | 1.27 (1.09-1.46) |
| Taiwan (Province of China) | 0.32 (-0.68-1.33) | -0.43 (-1.4-0.54) |
| Tajikistan | 9.12 (8.78-9.46) | 8.83 (8.45-9.21) |
| Thailand | 2.83 (2.71-2.94) | 2.57 (2.47-2.68) |
| Timor-Leste | 1.8 (1.57-2.03) | 1.59 (1.3-1.87) |
| Togo | 0.87 (0.67-1.07) | 0.82 (0.62-1.02) |
| Tokelau | 1.52 (1.39-1.66) | 1.5 (1.38-1.62) |
| Tonga | 1.95 (1.52-2.38) | 2.09 (1.66-2.52) |
| Trinidad and Tobago | -2.4 (-3.44--1.35) | -2.52 (-3.57--1.46) |
| Tunisia | 2.69 (2.5-2.89) | 2.73 (2.55-2.9) |
| Turkey | 2.61 (2.02-3.22) | 2.37 (1.73-3.02) |
| Turkmenistan | 8.54 (7.62-9.47) | 9.45 (8.36-10.55) |
| Tuvalu | 1.39 (1.27-1.51) | 1.42 (1.32-1.52) |
| Uganda | 2.84 (2.57-3.12) | 2.86 (2.58-3.15) |
| Ukraine | 4.76 (4.28-5.25) | 5.13 (4.52-5.75) |
| United Arab Emirates | 2.27 (1.67-2.86) | 2.35 (1.8-2.9) |
| United Kingdom | 5.97 (5.71-6.24) | 5.85 (5.58-6.12) |
| United Republic of Tanzania | 2.69 (2.51-2.87) | 2.96 (2.76-3.16) |
| United States of America | 5.56 (5.11-6.01) | 5.6 (5.16-6.05) |
| United States Virgin Islands | 0.31 (-0.21-0.85) | 0.36 (-0.18-0.91) |
| Uruguay | 6.1 (5.72-6.49) | 6.18 (5.81-6.56) |
| Uzbekistan | 13.72 (12.53-14.92) | 14.2 (12.89-15.52) |
| Vanuatu | 2.16 (2.08-2.24) | 2.31 (2.22-2.41) |
| Venezuela (Bolivarian Republic of) | -1.16 (-2.47-0.17) | -1.32 (-2.67-0.05) |
| Viet Nam | 0.99 (0.83-1.15) | 0.75 (0.59-0.91) |
| Yemen | 2.52 (2.37-2.67) | 2.44 (2.3-2.58) |
| Zambia | 1.1 (0.74-1.47) | 1.13 (0.75-1.51) |

NASH: nonalcoholic steatohepatitis; EAPCs: Estimated annual percentage changes; DALYs: disability-adjusted life years.

Fig. S1. The deaths of NASH-related liver cancer attributable to HFPG in countries and territories. (A) The ASR of NASH-related liver cancer deaths attributable to HFPG in 2019; (B) The relative change in percentage of NASH-related liver cancer deaths attributable to HFPG between 1990 and 2016. ASR, age-standardized rate.


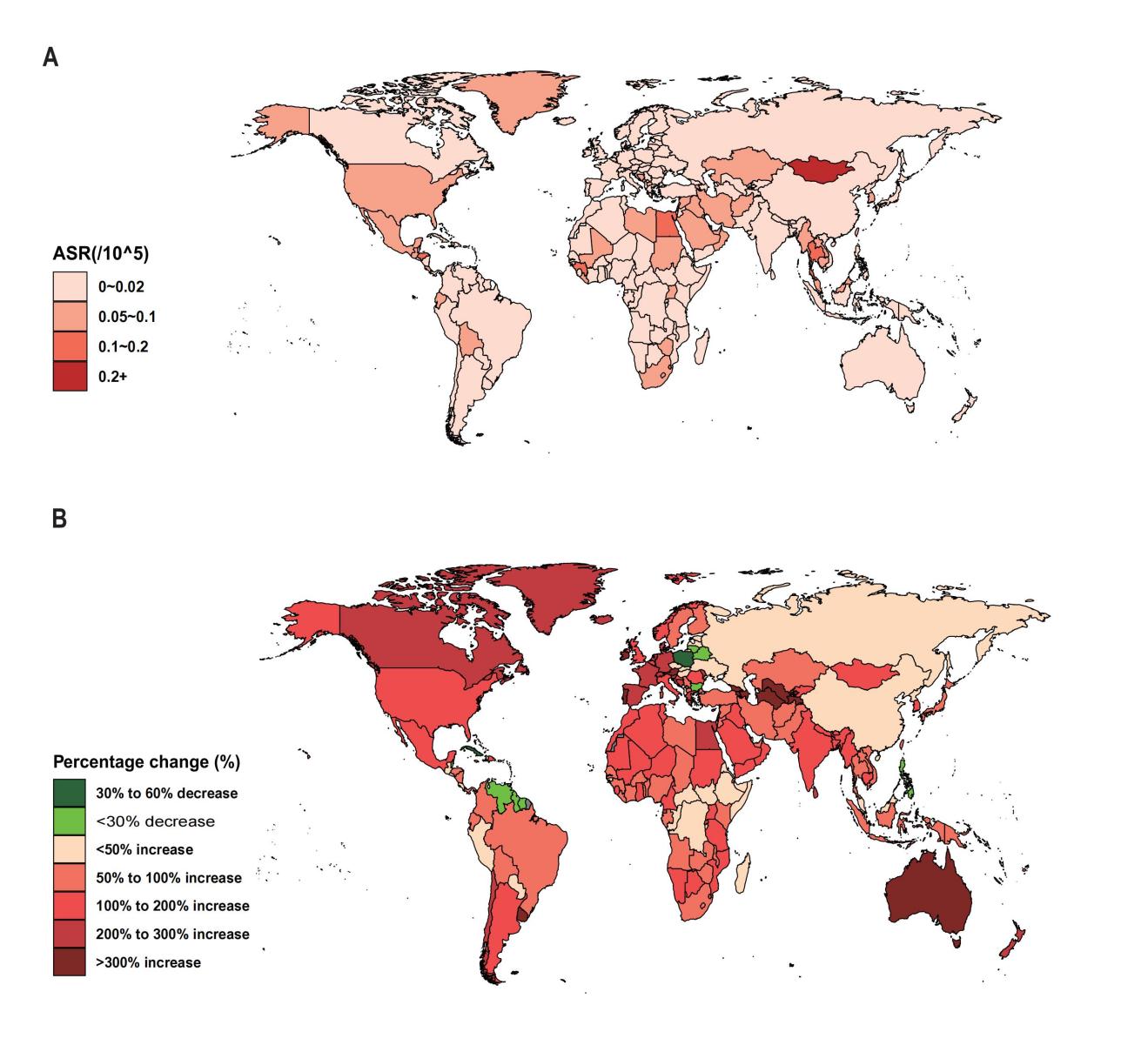


Fig. S2. The DALYs of NASH-related liver cancer attribute to HFPG in countries and territories. (A) The ASR of DALYs of NASH-related liver cancer attributable to HFPG in 2019; (B) The relative change in percentage of NASH-related liver cancer DALYs attributable to HFPG between 1990 and 2016. ASR, age-standardized rate. DALYs, disability adjusted life years.


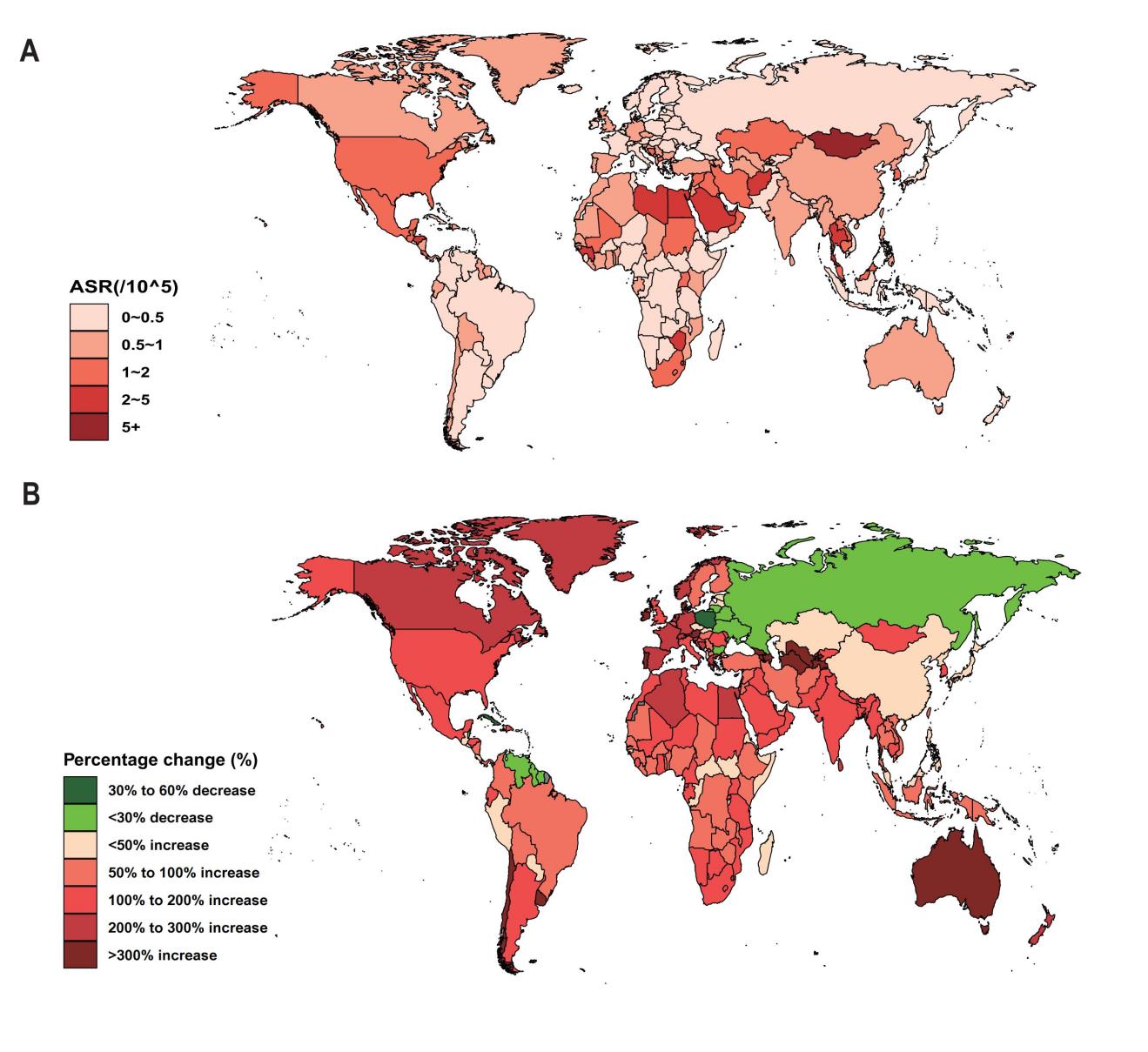


Fig. S3. The clusters of countries and territories in terms of the temporal trends of the NASH-related liver cancer burden attributable to HFPG.


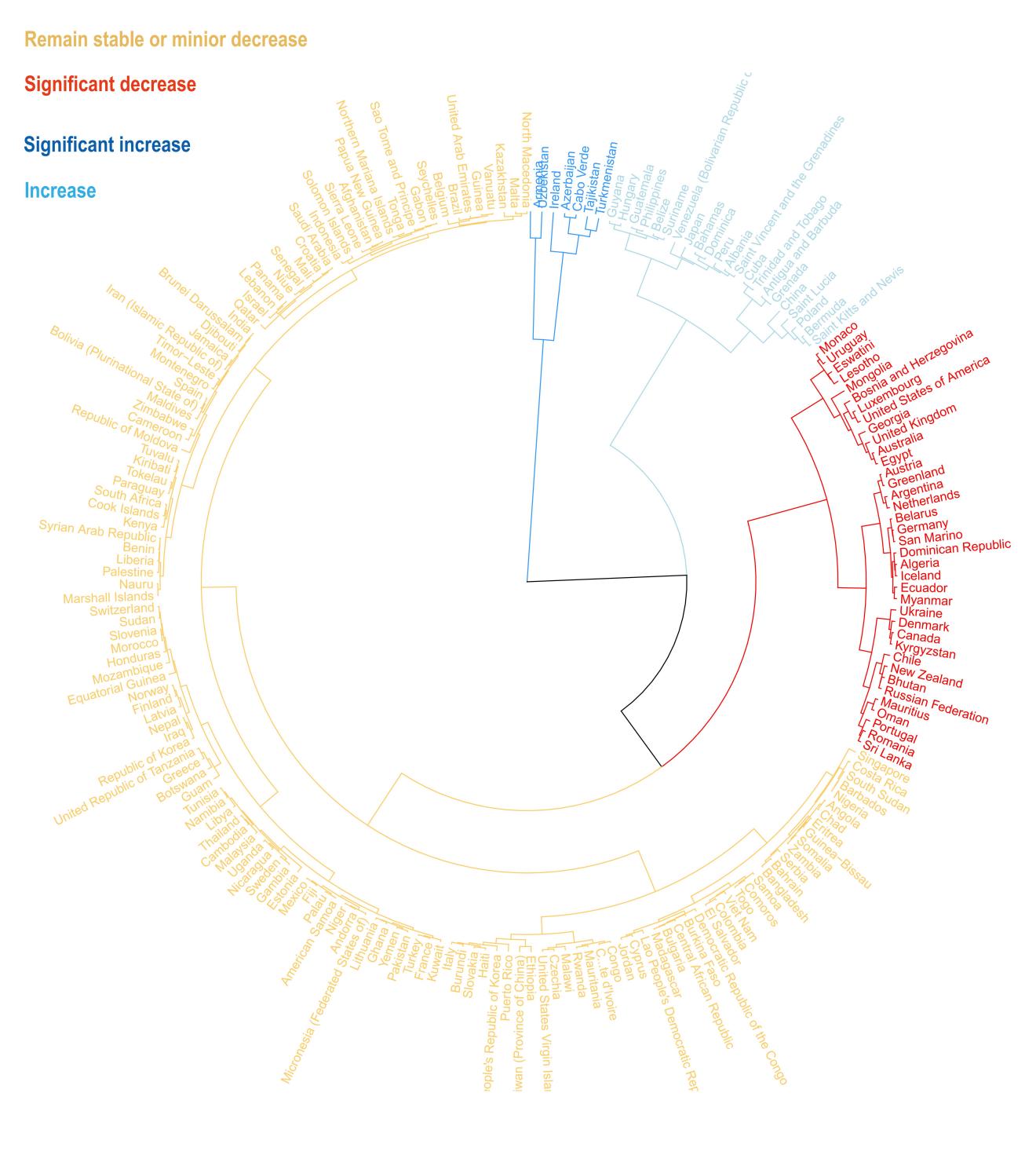


Fig. S4: Temporal trend of deaths rate of NASH-related liver cancer attributable to HFPG for different age group, 1990–2019.


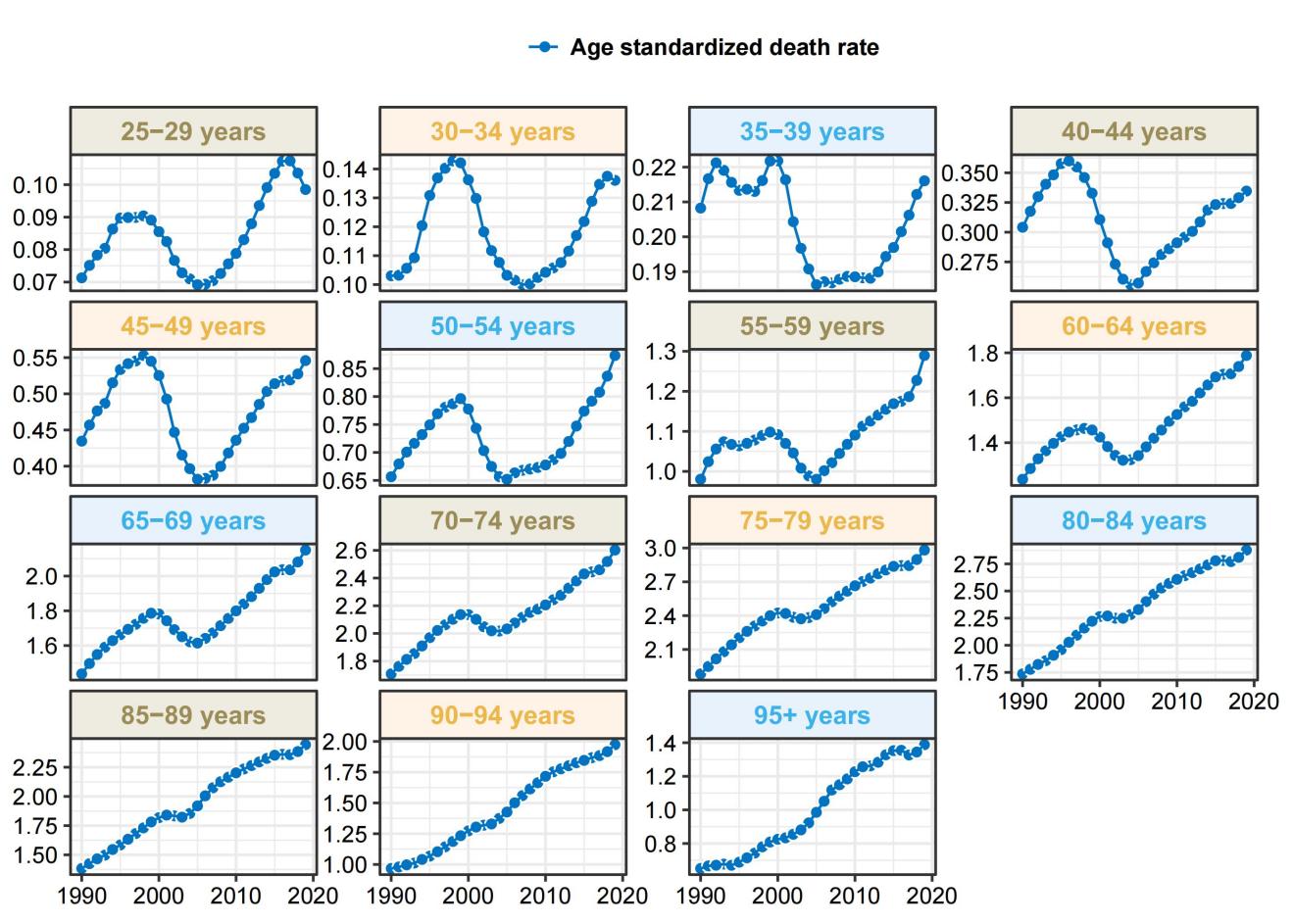


Fig. S5 Temporal trend of DALYs rate of NASH-related liver cancer attributable to HFPG for different age group, 1990–2019. DALYs, disability adjusted life years.


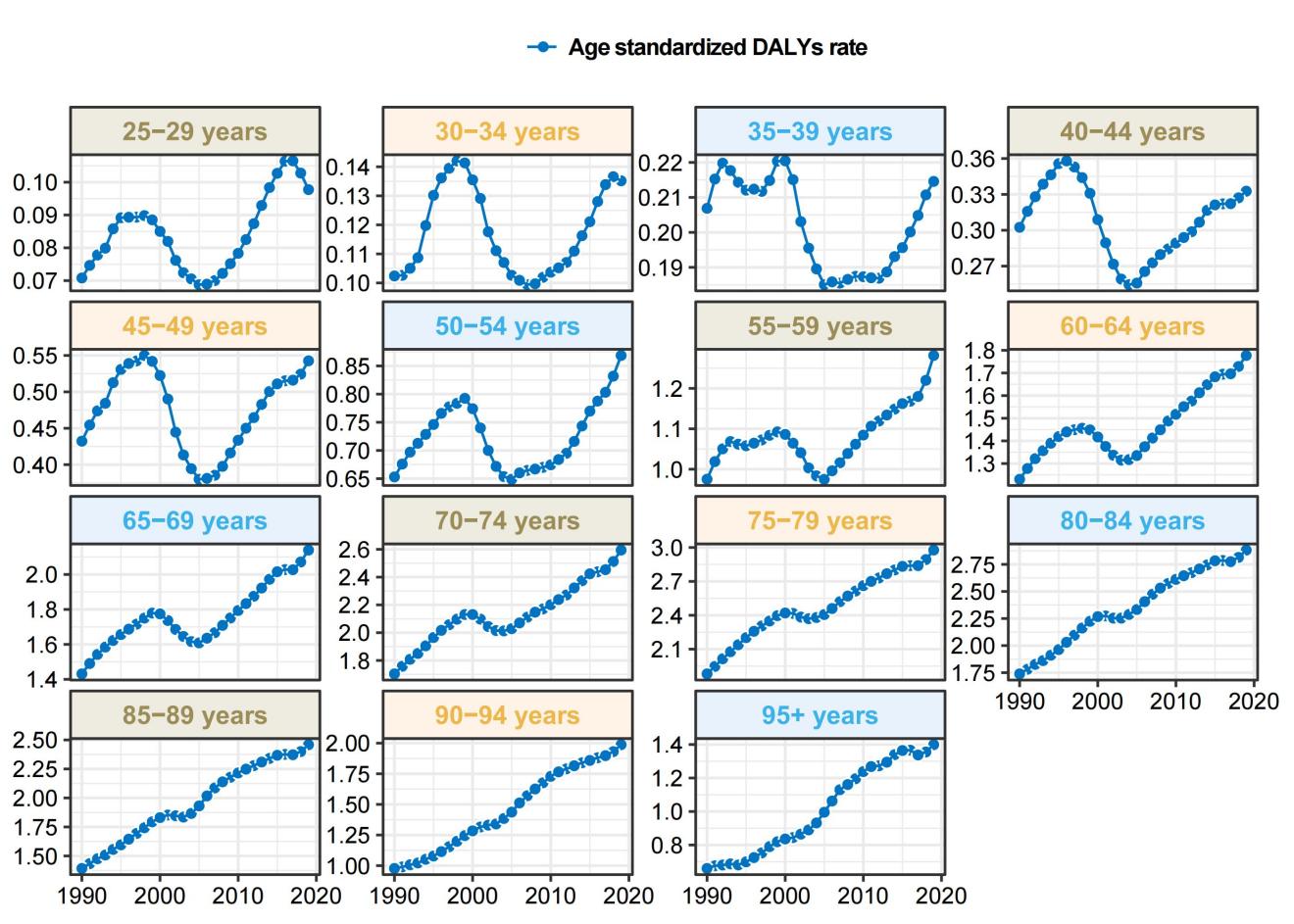


Fig. S6: Age-standardised rates of the burden of NASH-related liver cancer attributable to HFPG for locations by SDI, 1990–2019. (A) Age-standardised deaths rates of NASH-related liver cancer attributable to HFPG for regions by SDI, 1990–2019. (B) Age-standardised deaths rates of NASH-related liver cancer attributable to HFPG for nations and territories by SDI, 1990–2019. (C) Age-standardised DALYs rates of NASH-related liver cancer attributable to HFPG for regions by SDI, 1990–2019. (B) Age-standardised DALYs rates of NASH-related liver cancer attributable to HFPG for nations and territories by SDI, 1990–2019.


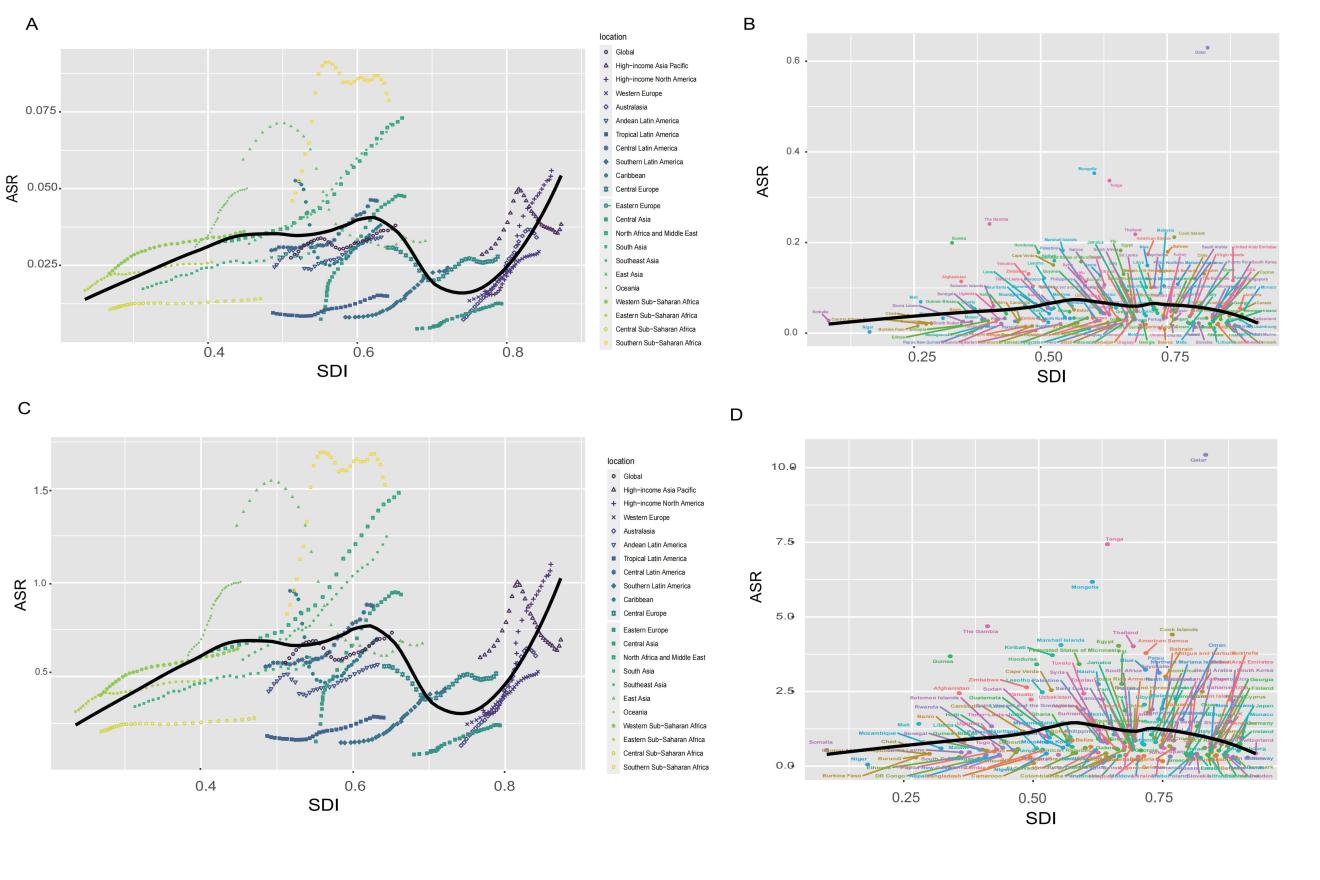


Fig. S7: The correlation between EAPC and ASR in 2019. (A) The correlation between EAPC and ASR of NASH-related liver cancer deaths attributable to HFPG in 2019. (B) The correlation between EAPC and ASR of NASH-related liver cancer DALYs in 2019 The circles represent countries that were available on data. The size of circle is increased with the cases of NASH-related liver cancer. NASH: nonalcoholic steatohepatitis; HFPG: high fasting plasma glucose; ASR, age-standardized rate; EAPC, estimated annual percentage change.


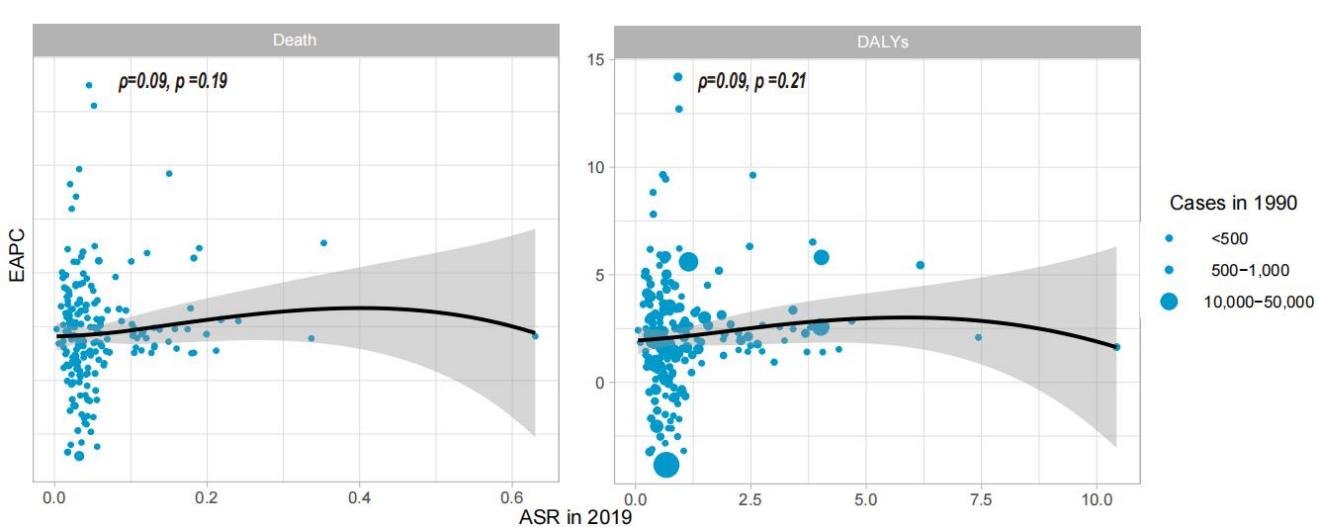


Fig. S8: The correlation between EAPC and SDI, HAQ or HDI. (A) The correlation between EAPC and SDI in 2019. (B) The correlation between EAPC and HAQ in 2019. (C) The correlation between EAPC and HDI in 2019 The circles represent countries that were available on data. SDI: socio-demographic index; HAQ: Healthcare Access and Quality; HDI:Human Development Index;EAPCs: Estimated annual percentage changes.


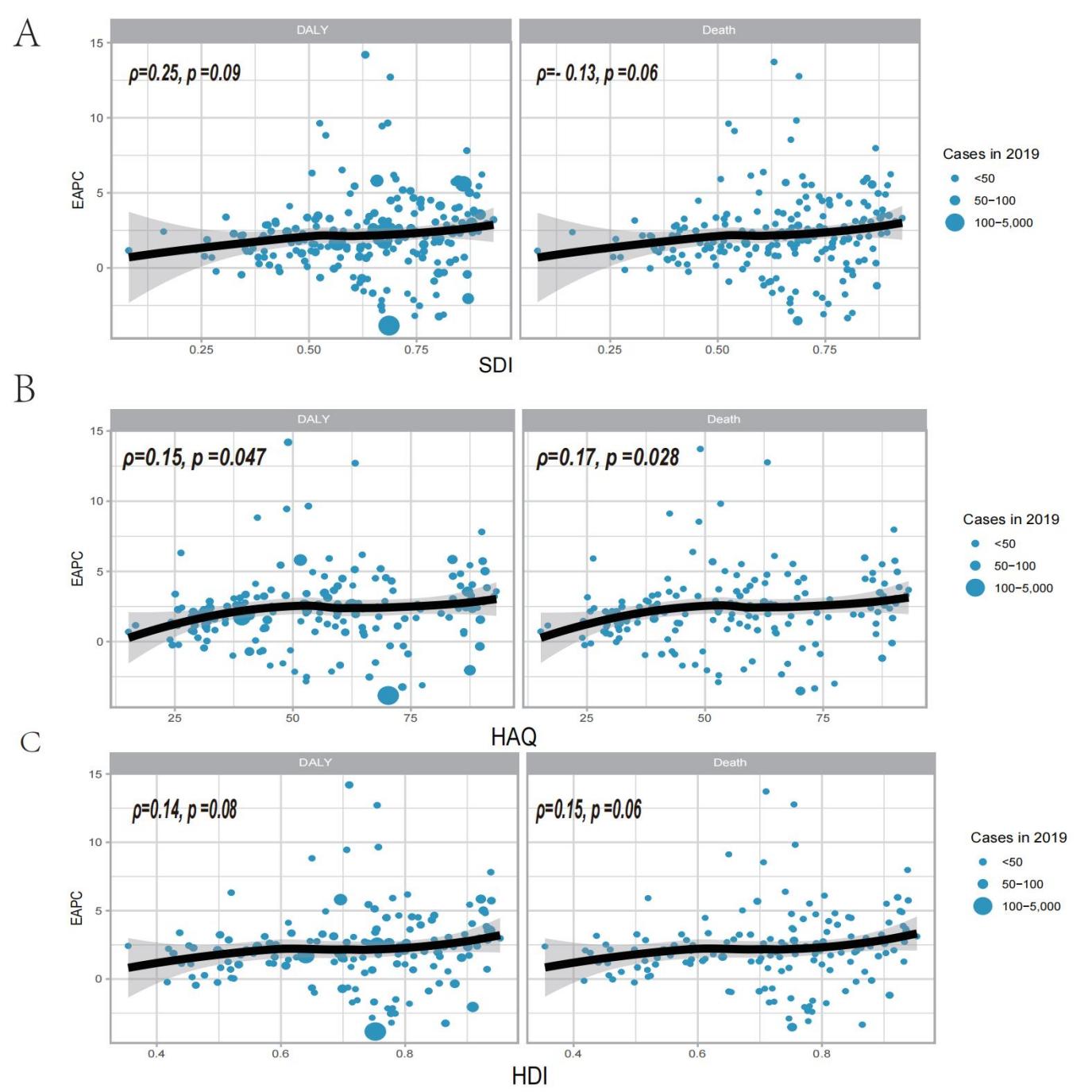

Supplement: Supplementary file 1 — Additional file 1:Table S1. Deaths of NASH-related liver cancer attributable to HFPG in 1990 and 2019, and its temporal trends from 1990 to 2019. Table S2. YLDs of NASH-related liver cancer attributable to HFPG in 1990 and 2019, and its temporal trends from 1990 to 2019. Table S3. YLLs of NASH-related liver cancer attributable to HFPG in 1990 and 2019, and its temporal trends from 1990 to 2019. Table S4. Deaths and DALYs of NASH-related liver cancer attributable to HFPG in countries and territories. Table S5. EAPC of NASH-related liver cancer deaths and DALYs attributable to HPFG in countries and territories. Figure. S1. The deaths of NASH-related liver cancer attributable to HFPG in countries and territories. (A) The ASR of NASH-related liver cancer deaths attributable to HFPG in 2019; (B) The relative change in percentage of NASH-related liver cancer deaths attributable to HFPG between 1990 and 2016. ASR, age-standardized rate. Figure. S2. The DALYs of NASH-related liver cancer attribute to HFPG in countries and territories. (A) The ASR of DALYs of NASH-related liver cancer attributable to HFPG in 2019; (B) The relative change in percentage of NASH-related liver cancer DALYs attributable to HFPG between 1990 and 2016. ASR, age-standardized rate. DALYs, disability adjusted life years. Figure. S3. The clusters of countries and territories in terms of the temporal trends of the NASH-related liver cancer burden attributable to HFPG. Figure. S4: Temporal trend of deaths rate of NASH-related liver cancer attributable to HFPG for different age group, 1990–2019. [file 13098_2022_976_MOESM1_ESM.docx]
